# Characterization of *carABpyrB* operon and role of *pyrE* in *Francisella novicida* biofilm

**DOI:** 10.1002/mbo3.70355

**Published:** 2026-09-07

**Authors:** Katie C. Sprinkel, Annika R. Geiger, Monique L. van Hoek

**Affiliations:** ^1^ School of Systems Biology George Mason University Manassas Virginia USA; ^2^ Center for Infectious Disease Research George Mason University Manassas Virginia USA

**Keywords:** biofilm, *Francisella novicida*, pyrimidine biosynthesis, uracil

## Abstract

Pyrimidine biosynthesis is essential for bacterial growth, but its role in regulating biofilm formation in *Francisella (F.) novicida* remains poorly defined. In this study, we experimentally defined the *carABpyrB* operon in *F. novicida* and investigated how disruption of the de novo pyrimidine biosynthesis pathway affects growth and biofilm formation under nutrient‐restricted conditions. Reverse transcriptase PCR confirmed co‐transcription of *carA*, *carB*, and *pyrB*, and promoter prediction identified two putative σ^70^‐dependent promoter regions upstream of *carA*. Transposon mutants disrupted in *carA*, *carB*, and *pyrB* exhibited pronounced growth defects in Chamberlain's Defined Medium that were restored by uracil supplementation, confirming pyrimidine auxotrophy and functional disruption of de novo pyrimidine biosynthesis. We then extended this analysis to additional genes in the pyrimidine biosynthetic pathway and assessed biofilm formation in modified Mueller–Hinton broth, a nutrient‐restricted condition. In this medium, *carA*, *carB*, *pyrB*, and *pyrE* mutants exhibited growth deficiencies; however, the *pyrE* mutant uniquely produced significantly more biofilm than the wild type. This phenotype remained evident even without growth normalization, with the *pyrE* mutant producing 3.3‐fold more biofilm than wild type, despite impaired growth, and increased to 11.8‐fold when normalized to growth. Quantitative PCR demonstrated that uracil supplementation represses *carA*, *carB*, and *pyrB* transcription, consistent with feedback regulation of the pathway. Together, these findings indicate that pyrimidine limitation is not simply a growth‐limiting condition but can alter biofilm regulation, with *pyrE* disruption revealing a strong association between de novo pyrimidine biosynthesis and biofilm formation.

## Graphical abstract

Pyrimidine biosynthesis links metabolism to biofilm regulation in *Francisella novicida*. During nutrient limitation, reduced pyrimidine availability shifts metabolism from uracil salvage to de novo synthesis, increasing biofilm formation. Mutants in *carABpyrB* exhibit uracil‐dependent growth defects and elevated biofilm:growth ratios, revealing pyrimidine limitation as a key metabolic cue for biofilm regulation.

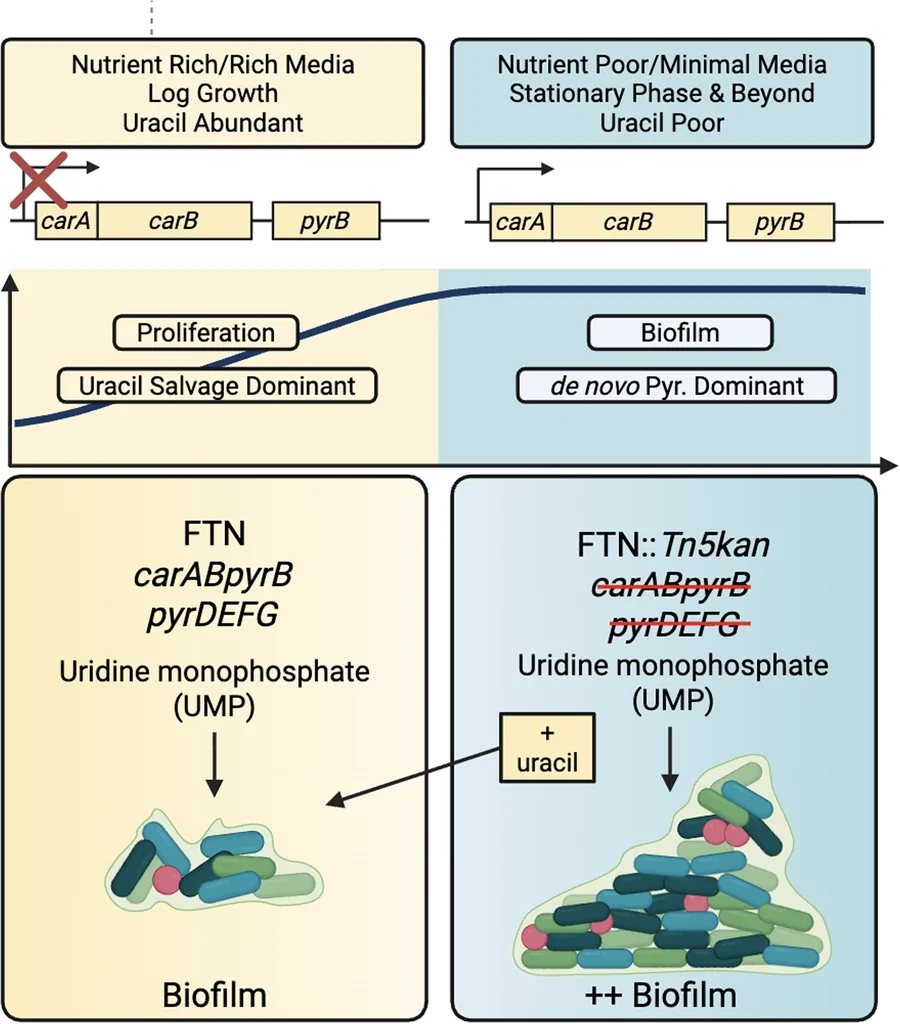

## 1 Introduction

**Figure 1:**
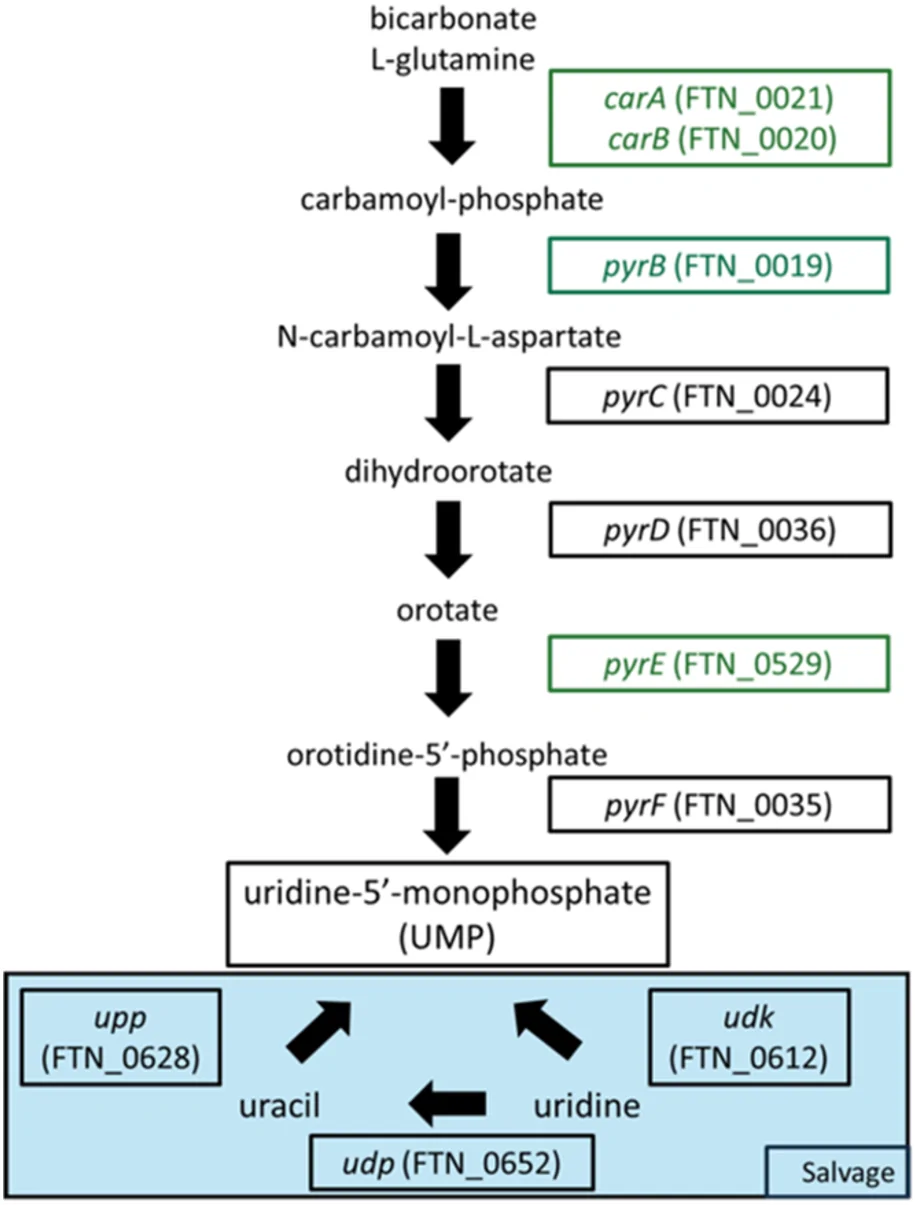
The pathway in *Francisella novicida* that produces the pyrimidine nucleotide precursor, uridine monophosphate (UMP). The de novo pyrimidine biosynthesis pathway with corresponding enzymes is shown; gene locus numbers for *F. novicida* are shown. The salvage pathway genes that have been detected in *Francisella* are highlighted in blue.

Carbamoyl‐phosphate synthetase (CPS) catalyzes the conversion of bicarbonate, ATP, and glutamine to carbamoyl phosphate (CP), which serves as a precursor for both arginine and pyrimidine biosynthesis pathways in many organisms. The bacterial CPS holoenzyme consists of two subunits together in a complex: the smaller subunit CPSI, encoded by the *carA* gene, and the larger subunit CPSII, encoded by *carB* (Thoden et al. 2002) (Figure 1). The pyrimidine pathway ultimately synthesizes uridine monophosphate (a nucleotide) from L‐glutamine through a series of six enzymatic reactions starting with the hydrolysis of L‐glutamine by the subunit encoded by *carA*, releasing ammonia and glutamate. The ammonia formed in the hydrolysis of glutamine is used by CarB, in addition to bicarbonate and ATP, to produce CP (Thoden et al. 2002; Charlier et al. 1988). PyrB encodes aspartate carbamoyl transferase, an enzyme that catalyzes the condensation of aspartate with CP, generating *N*‐carbamoyl aspartate. This committed reaction is the second enzymatic step in the biosynthesis of pyrimidines. The three species of *Francisella (F.)* bacteria that are commonly studied include: *Francisella tularensis* subsp. *tularensis* Schu S4, the fully virulent Type A strain that infects humans and animals, *Francisella tularensis* subsp. *holarctica* live vaccine strain (LVS), the attenuated Type B strain that was developed as a vaccine, and *Francisella tularensis* subsp. *novicida*, an environmental, non‐pathogenic strain that is 98% identical to the fully virulent strain. In *Francisella novicida*, the operon for carbamoyl synthesis has not been formally defined. Qin and Mann (2006) proposed in SchuS4 that the *carAB* operon also includes a third gene, *pyrB*, adjacent to *carAB* in all strains of *Francisella*, which we experimentally confirmed in this study. Regulation of the *carAB* operon in bacterial species has shown dependence on metabolic feedback from end‐products such as uracil. Mutants in the de novo pyrimidine biosynthesis pathway can often be functionally complemented by exogenous uracil, provided the salvage pathway is intact. This complementation occurs through conversion of uracil to uridine monophosphate by uracil phosphoribosyltransferase (UPRT, FTN_0628), thereby bypassing upstream biosynthetic steps (Figure 1) (Moffatt and Ashihara 2002; Bosch et al. 2020; Meibom and Charbit 2010).

### 1.1 *F. tularensis* subsp. *tularensis* Schu S4

The *carAB* genes were not identified as being required for in vitro growth in *F. tularensis* subsp. *tularensis* Schu S4 in rich medium (Ireland et al. 2019). However, *F. tularensis* Schu S4 mutants with deficiencies in early steps of the carbamoyl synthesis pathway, particularly in transposon mutants lacking functional *carA*, *carB* or *pyrB* genes in Schu S4 showed reduced growth in HepG2 cells, but not in J774A.1 macrophages (consistent with the cell‐type specific requirements for pyrimidine biosynthesis) (Qin and Mann 2006; Schulert et al. 2009). In mice, the *pyrB* mutant was attenuated. A screen of Schu S4 mutants did not identify an attenuated phenotype for *pyrC*, *pyrD*, *pyrE*, or *pyrF* mutants (Qin and Mann 2006; Horzempa et al. 2010). Although mutants lacking these genes can not replicate in macrophages (Qin and Mann 2006, Horzempa et al. 2010) or neutrophils (Schulert 2009) due to the lack of intracellular uracil, the *delta‐pyrF* strain retained its virulence in animal models by replicating in non‐macrophage cells (Horzempa et al. 2010).

### 1.2 *F. tularensis* subsp. *holarctica* LVS

Horzempa et al. (2008) found that *pyrB*, *carA*, and *carB* were induced in expression (2.2–2.6 fold) when LVS growth was shifted to 37°C. Screening of transposon mutants of *F. tularensis* subsp. *holarctica* LVS in human polymorphonuclear leukocytes and macrophages identified carbamoyl phosphate pathway mutant *carA*, *carB*, and *pyrB* as being unable to inhibit oxidative burst responses in neutrophils (Schulert et al. 2009). A Himar1‐based transposon mutagenesis study found that while *carA* and *pyrD* mutants were auxotrophic for uracil, they were indistinguishable from wild type when measuring replication in J774A.1 macrophage (Maier et al. 2006). In contrast, Su et al. (2007) did not report any pyrimidine biosynthesis genes in their genome‐wide virulence screen of LVS in the BALB/c mouse intranasal infection model with their custom transposon library, indicating that these loci were not implicated as virulence determinants in vivo for LVS (Su et al. 2007). To analyze the complete transcriptome of *F. tularensis* LVS during phagosomal escape and cytosolic replication within murine macrophages, Bent et al. (2013) used a capture‐based bacterial transcript enrichment strategy. The 4 h post‐infection time point represents a transition state where some bacteria remained within phagosomes and others escaped into the cytosol, while the 8 h post‐infection time point represented most bacteria in the cytosol. They found that *carA* was significantly upregulated after 4 h but not after 8 h of infection. There was no significant expression change in *carB*, *pyrB*, *pyrC*, *pyrD*, *pyrE*, or *pyrF* at either time point (Bent et al. 2013). The requirement for the pyrimidine pathway genes was not demonstrated in the P388D1 murine macrophage model, similar to what was shown for *F. tularensis* and J774A.1 murine macrophages (Qin and Mann 2006; Horzempa et al. 2010). Macrophage cells may produce sufficient uracil to overcome the auxotrophy requirement. A genome‐wide ChIP‐seq mapping in *F. tularensis* LVS identified *carA* and *pyrF* as direct targets of the MglA–SspA–PigR virulence regulator complex (Ramsey et al. 2015), suggesting that these pyrimidine biosynthesis genes are part of the PigR‐regulon, and that *F. tularensis* LVS coordinates nucleotide metabolism with other virulence‐associated functions.

### 1.3 *F. novicida* U112

In *F. novicida*, genes in the carbamoyl phosphate pathway appear to be important for intracellular replication in macropahges unlike with other *Francisella* strains, and also in vivo. Weiss et al. (2007) screened a *F. novicida* transposon mutant library intraperitoneally in C57BL/6 mice and found that recovery, including *carA*, *carB*, *pyrB, and pyrF*, was diminished, suggesting that these genes are essential for *Francisella*'s replication and survival in vivo (Weiss et al. 2007). In a follow‐on study, Llewellyn et al. (2011) found that *F. novicida carA, carB*, and *pyrB* mutants had decreased intracellular replication in RAW 264.7 murine macrophages. The *pyrC* deletion mutant in *F. novicida* had less replication than wild‐type and contributes to immunosuppression and STING pathway activation in U937 cells by inducing TNF‐alpha, interleukin‐1‐beta and interferon‐beta production (Nakamura et al. 2022). Additional studies showed that *F. novicida* transposon insertion mutants in pyrB and carB exhibited impaired growth in human U937 macrophages (Asare and Abu Kwaik 2010). The CarA, CarB and PyrB proteins were identified in Francisella outer membrane vesicles (OMVs) through a proteomics study of *F. novicida* and *F. philomiragia* OMVs (Pierson et al. 2011).

Tempel et al. (2006), found that their in‐house generated *carB* transposon mutants of *F. novicida* were attenuated in BALB/c mice following an intraperitoneal infection with a normally lethal dose resulting in no signs of severe illness and 100% survival in the mice (Tempel et al. 2006). However, Kraemer et al. (2009) did not identify any mutants in this pathway in their study of *F. novicida* mutants delivered via aerosol infection, using the Gallagher et al. *F. novicida* transposon mutant library (also used in this study [Gallagher et al. 2007]) revealing a role for the route of infection. Ahlund et al. (2010) showed that carAB and pyrBCFD were essential for *F. novicida* lethality when these mutants were injected directly into *Drosphila melanogaster*. These findings reveal that the contribution of these genes to virulence is complex and context‐dependent, varying with the host cell type or the route of infection (e.g., intraperitoneal vs. aerosol).

The importance of pyrimidine biosynthesis for *F. novicida* growth in vitro is further supported by genotype‐phenotype studies. Enstrom et al. (2012) conducted a comprehensive in vitro analysis of the Gallagher et al. transposon mutant library to identify genes essential for fitness in rich and minimal media (Enstrom et al. 2012). They found that multiple genes involved in the de novo pyrimidine biosynthesis pathway (including *carA*, *carB*, and *pyrB*) were conditionally essential for *F. novicida* growth in nutritionally limited or stressful conditions (conditional auxotrophy) while growing normally in rich media.

### 1.4 Role of *carAB* and *pyrB* in *Francisella* Pathogenesis

Evidence from these studies demonstrates that *carA*, *carB*, and *pyrB* are essential for *Francisella* survival in host environments: mutants grow normally in rich broth and some immortalized cell lines but are markedly attenuated in primary human monocytes and monocyte‐derived macrophages, fail to suppress the neutrophil oxidative burst, and are underrepresented in in vivo negative‐selection screens, indicating loss of fitness during infection. These studies show that the contribution of these genes to pathogenesis is context dependent, becoming critical under the nutrient‐limited conditions of macrophages or other host tissues, where they may act as in vivo fitness determinants that enable *F. tularensis* to meet pyrimidine demands and sustain intracellular replication and disease progression (Joshi et al. 2026). Consistently, *Francisella* exhibits a strong metabolic dependency on pyrimidine biosynthesis under nutrient limitation. Together, these data suggested the hypothesis that pyrimidine biosynthesis supports basic growth requirements but may also modulate *F. novicida* biofilm production through nutrient‐responsive pathways or metabolic signaling.

### 1.5 Biofilms of *F. novicida*

Biofilms are multicellular bacterial communities encased in a self‐produced extracellular matrix that confer protection against environmental stressors, antimicrobial agents, and immune defenses. *F. novicida* biofilm formation is a complex process regulated by both environmental and intracellular signals. Two‐component regulatory systems, including orphan sensor kinase *qseC* and response regulator *qseB*, have been shown to be essential elements in controlling biofilm production (Durham‐Colleran et al. 2010; van Hoek 2013; Dean and van Hoek 2015; van Hoek et al. 2019). These regulatory systems may respond to environmental signals to modulate gene expression related to biofilm production. Mutants in the pyrimidine synthesis pathway, especially of *carAB*, in *Xanthomonas* and *Pseudomonas* bacteria display significant changes to their phenotype, including increased biofilm formation and decreased virulence (Zhuo et al. 2015; Butcher et al. 2016). In *Francisella*, biofilm formation is thought to play a crucial role in its environmental persistence, particularly in water and soil environments (Durham‐Colleran et al. 2010; van Hoek 2013; Dean and van Hoek 2015; van Hoek et al. 2019; Chung et al. 2014; Dean et al. 2015, 2020; Verhoeven et al. 2010). Pathogenic *F. tularensis* Schu S4 and the environmental strain *F. novicida* U112 (sharing 98.1% nucleotide identity [Rohmer et al. 2007]) have both been shown to form biofilms, still potentially supporting their survival during their life cycle stage outside a host (Durham‐Colleran et al. 2010; Dean and van Hoek 2015; Chung et al. 2014; Dean et al. 2009, 2015, 2020). Biofilms are thought to enable *Francisella* to withstand unfavorable environmental conditions, facilitating its persistence in natural reservoirs (Durham‐Colleran et al. 2010; van Hoek 2013; Verhoeven et al. 2010). We characterized the effect of mutants in the pyrimidine biosynthesis pathway and uracil on biofilm formation in *F. novicida*. Understanding the molecular mechanisms regulating biofilm formation in *Francisella* is important for unraveling its environmental survival strategies.

## 2 Materials and Methods

### 2.1 Bacterial Strains

Wild‐type *F. novicida* (Larson et al. 1955; Olsufiev et al. 1959) was obtained through BEI Resources, NIAID, NIH: *F. tularensis* subsp. *novicida*, Strain Utah 112, NR‐13, formerly ATCC 15482. The *F. novicida* transposon mutant library used in this study was acquired through BEI Resources, NIAID, NIH: *F. tularensis* subsp. *novicida*, “Two‐Allele” Transposon Mutant Library, Plates 1–33 (NR‐8034, BEI Resources, NR‐52248, Manassas, VA, USA; Table S1) (Gallagher et al. 2007). All bacterial strains were cultured in tryptic soy broth supplemented with 0.1% cysteine (TSB‐C) or freshly made, and stored cold in the dark, Mueller–Hinton broth (MHB) supplemented with 0.025% ferric pyrophosphate, 0.02% IsoVitaleX, and 0.1% glucose as a carbon source (modified Mueller–Hinton Broth [MMHB]) (Margolis et al. 2010) unless otherwise specified. Cultures were grown in a shaking incubator at 37°C. For these studies, the growth was typically for 48 h to form a biofilm. Some biofilm experiments were done in MMHB or TSB‐C, see details below and in Supplemental data. For auxotrophy and gene regulation experiments, cells were grown in Chamberlain's Defined Medium (CDM) (Chamberlain 1965), a minimal medium that supports the fastidious growth requirements of *Francisella*.

### 2.2 Genomic Information

The relative location of the genes in different species of *Francisella* was studied using the PATRIC website, https://www.bv‐brc.org/ (Wattam et al. 2013) (Figure S1, Table S2).

### 2.3 Promoter Prediction Tools

Putative promoter sequences and transcription start sites (TSSs) within the *F. novicida* genome were identified using BPROM program from Softberry (Solovyev and Salamov 2011). The *F. novicida* reference genome NC_008601.1 (c22992‐22500) was used. There 100% sequence homology to the *F. novicida* reference genome CP009633.1 in this region.

Promoters were hypothetically predicted with BPROM, a promoter recognition program that recognizes bacterial σ^70^ sites in *E. coli*. The BPROM model uses Linear Discriminant Analysis (LDA) to predict potential bacterial promoters. The model predicts the presence of a promoter based on five relatively conserved motifs found in known promoters in *Escherichia coli*, so there may be some differences to Francisella species. The BPROM algorithm uses the LDA to discriminate between promoter and non‐promoter sequences (Softberry) (Solovyev and Salamov 2011). We analyzed the *carA*–upstream intergenic region of *F. novicida* U112 (*E. coli* σ^70^ predictor; default settings). The input was a 492 bp fragment (NC_008601.1 [c22992‐22500]) that spans the sequence immediately upstream of FTN_0021, including the *carA* ATG. BPROM predicts (−35/−10) promoter elements, but not ribosome binding sites. The Linear Discriminant Function (LDF) score (decision threshold 0.20) reflects the classifier's confidence; higher scores indicate stronger σ^70^promoter–like features, BPROM reports positions as 1based coordinates relative to the leftmost base of the query sequence and provides a linear discriminant score (LDF) plus a score for the inferred −35/−10 hexamers. Two σ^70^ promoters were predicted in this window: The first is called P1 (high confidence): Promoter position 88; LDF = 8.76. The −10 box, TTATAGATT at position 73 (score = 38); the −35 box, TTTAAA at 50 (score = 41). The −35/−10 spacing (end of −35 to start of −10) is 17 bp, consistent with a canonical σ^70^ promoter. The second is called P2 (lower confidence): Promoter position 385; LDF = 1.77. The −10 box, TTTCAAAAT at 369 (score = 53); −35 box, ATGACA at 355 (score = 36). The −10 sequence may be an extended/degenerate variant; the overall spacing falls within a relaxed σ^70^ range. For the analyses and figures, we labeled the higher‐scoring site as **P1** and the weaker site as **P2** and mapped their positions back to the *carA* start to depict −35 boxes, −10 boxes, and ATG in scale. All coordinates and motif sequences are reported as returned by BPROM for this 425 bp query. The Ramsey et al. (2015) high‐confidence σ^70^ peak in *F. tularensis* LVS that is ~40 bp upstream of *carA* is approximately equivalent to position 382 (**T**) in the *F. novicida* sequence shown (Ramsey et al. 2015), suggesting good correlation of the prediction. The same regions of *F. novicida* and *F. tularensis* LVS were scanned with ProPr v2.0 (http://ppp.molgenrug.nl/) for additional analysis (de Jong et al. 2012) yielding similar results.

### 2.4 Identification of Potential σ^70^‐Binding Site Upstream of *carA* in *F. novicida U112*

The *F. tularensis* LVS *carA* promoter region was defined according to the TSS mapping and σ^70^ ChIP‐seq analysis of Ramsey et al. (2015). who experimentally determined the location of the σ^70^‐binding site upstream of the *F. tularensis* LVS *carA* (FTL_0030) gene. The genomic region was retrieved from NCBI GenBank accession NC_007880.1 (coordinates 31,790–32,440). The corresponding promoter region upstream of *carA* (FTN_0020) in *F. novicida* was identified and retrieved from NCBI GenBank accession CP009633.1 (coordinates 196,928–197,578). Sequences were extracted in FASTA format and aligned in Jalview v2.11.4.1 using the FFT‐NS‐i algorithm implemented in MAFFT (Katoh and Standley 2013), which applies iterative refinement with two cycles to optimize alignment accuracy. The alignment was visually inspected to confirm conservation of the −35 and −10 promoter elements predicted in *F. novicida* and the position of the experimentally determined σ^70^‐binding site. The presence or absence of the PigR‐Response Element motif (TGCTGTA) was assessed in each sequence in the required position (6–7 bp upstream of the −35 element) according to the criteria of Ramsey et al. (2015).

### 2.5 RT‐PCR for Operon Determination and qPCR for *carAB* Operon Transcription and *pyrE/lpxH* Expression

Total RNA was extracted using TRIzol reagent (Invitrogen) following the manufacturer's instructions. Briefly, bacterial samples were lysed in TRIzol, and chloroform was added (1:5 ratio of chloroform to TRIzol). The mixture was vortexed, incubated at room temperature for 3 min, and centrifuged at 12,000×*g* for 15 min at 4°C. The aqueous phase containing RNA was transferred to a fresh tube and precipitated with an equal volume of isopropanol. After centrifugation (12,000×*g* for 10 min at 4°C), the RNA pellet was washed with 75% ethanol, air‐dried, and resuspended in RNase‐free water. RNA integrity and purity were assessed using a NanoDrop spectrophotometer (Thermo Fisher Scientific, Waltham, MA, USA). For RT‐PCR for operon determination, cDNA synthesis was performed from 500 ng of total RNA that was treated with DNase I (Qiagen) to remove genomic DNA contamination. Reverse transcription was performed using the QuantiTect Reverse Transcription Kit (Qiagen) according to the manufacturer's protocol. Briefly, RNA was mixed with gDNA Wipeout Buffer and incubated at 42°C for 2 min to eliminate genomic DNA. Reverse transcription was carried out in a 20 µL reaction containing Quantitect Reverse Transcriptase, RT Buffer, and RT Primer Mix, incubated at 42°C for 15–30 min, followed by enzyme inactivation at 95°C for 3 min. The synthesized cDNA was used immediately for PCR. PCR was performed using the Phusion High‐Fidelity DNA Polymerase Kit (New England Biolabs). Each 50 µL reaction contained 10 µL of 5× Phusion HF Buffer, 1 µL of dNTPs (200 µM each), 1 µL of forward primer (10 µM), 1 µL of reverse primer (10 µM), 1 µL of Phusion DNA Polymerase (2 U/µL), 2 µL of cDNA template, and nuclease‐free water to 50 µL. PCR conditions were: (1) Initial denaturation: 98°C for 30 s; (2) Cycling (30 cycles): denaturation at 98°C for 10 s, annealing at 64.5°C for 15 s, and extension at 72°C for 30 s; and (3) Final extension at 72°C for 5 min. PCR products were RT‐PCR analyzed by agarose gel electrophoresis and visualized on a UV transilluminator using ethidium bromide at a final concentration of 0.5 µg/mL. Primer sets are described in Table S3.

For qRT‐PCR to examine uracil regulation of *carAB* transcription, *F. novicida* U112 was grown for 20 h in TSB‐C with or without 12.5 µg/mL uracil. As cited above, RNA was extracted, and the Applied Biosystems Power SYBR RNA‐to‐CT 1‐Step Kit Protocol was used for reverse transcription and qPCR. Primer sets are described in Table S3.

### 2.6 Rescue of In Vitro Growth With Uracil in CDM

#### 2.6.1 Agar Plates

Suspensions of *F. novicida* U112 and *F. novicida* transposon mutants *carA‐1::Tn5kan*, *carA‐2::Tn5kan*, *carB‐1::Tn5kan*, *carB‐2::Tn5kan*, *pyrB‐1::Tn5kan*, and *pyrB‐2::Tn5kan* were grown overnight in TSB‐C or, for the transposon mutants, TSB‐C plus 10 µg/mL kanamycin, respectively. Overnight cultures were streaked onto CDM‐A with or without uracil (50 µg/mL), incubated at 37°C for 48 h, after which time plates were examined for growth, following Maier et al. (2006).

#### 2.6.2 Liquid Growth Curves

For growth curve analysis in CDM broth, bacterial cultures were initially grown overnight in TSB‐C (*F. novicida* U112) or TSB‐C plus 10 µg/mL kanamycin (*F. novicida* transposon mutants that showed uracil auxotrophy). Overnight suspension cultures were diluted to 0.5 McFarland and then further diluted 1:100 in fresh CDM for growth in 96‐well polystyrene plates (3 × 10^5^ CFU per well in 200 µL) (Falcon 353072). The microtiter plate was placed with its lid into the plate reader (Biotek Cytation 5), and 10 s shaking was performed before each OD600 nm reading at 15 min intervals for 72 h.

### 2.7 Crystal Violet Assay for Biofilm (96 Well Protocol)

Biofilm was measured as previously described (Durham‐Colleran et al. 2010; Dean et al. 2015; Dean and van Hoek 2015; Dean et al. 2020) with the following modifications. Bacteria were inoculated (1 × 10^6^ cells per well) in 200 μL of MMHB (or MMHB + 50 µg/mL uracil, for rescue experiments) in a 96‐well plate (Falcon 353072) and were incubated at 37°C for 48 h. Optical density (OD) of the cultures (OD600) was determined prior to staining as a measure of bacterial growth. Biofilm production was measured using the crystal violet (CV stain technique with absorbance measurements at 590 nm of the solubilized stain [CV590]) (Durham‐Colleran et al. 2010). Additional data is shown in Table S4.

### 2.8 Effect of Uracil Supplementation on Gene Expression in CDM

*F. novicida* was grown in CDM with or without uracil (12.5 µg/mL) following Maier et al. (2006). Expression of the *carAB* pathway in most organisms is feedback‐regulated by intracellular pyrimidine nucleotides (UMP, UTP, and CTP). Exogenous uracil is converted to UMP by uracil phosphoribosyltransferase (Upp), which increases intracellular pyrimidine pools and, in turn, modulates transcription of the biosynthetic genes in a concentration‐dependent manner. To prevent saturation artifacts associated with either uracil deprivation or full repression of the pathway, qPCR assays were performed using a sub‐saturating uracil concentration (12.5 µg/mL). Pilot titrations ranging from 12.5 µg/mL to 50 µg/mL produced graded, dose‐dependent changes in transcript levels, with 12.5 µg/mL providing a consistent midrange signal that remained sensitive to regulatory changes while minimizing growth‐related effects. Using a sub‐saturating concentration allowed transcript levels to be measured within the linear range of the regulatory response, preserving physiological relevance, and avoiding ceiling or floor effects typical of extreme metabolite conditions. After 12 h of growth, RNA was extracted using TRIzol. Primers spanning the intergenic regions of the genes of interest were designed to detect the full length of the mRNA. Relative quantification of gene expression was normalized to 16S rRNA. Statistical significance was determined by two‐way ANOVA; **p = 0.0043, ***p = 0.0003, and ****p < 0.0001.

## 3 Results

### 3.1 Experimental Demonstration of the *carABpyrB* Operon in *F. novicida*

**Figure 2:**
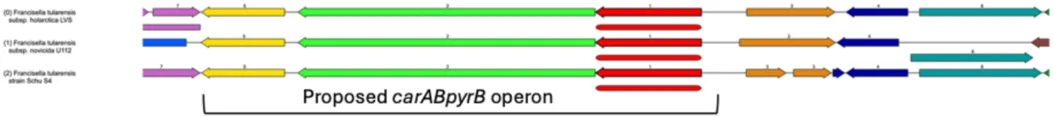
Organization of *carA, carB*, and *pyrB* in three *Francisella* strains. The red gene is *carA*, the bright green gene is *carB*, and the yellow gene is *pyrB*. In *Francisella tularensis* Schu S4 and *F. tularensis* LVS, the gene neighborhood begins to diverge outside of this proposed operon compared to *Francisella novicida*. Image generated from PATRIC (bv‐brc.org).

In *F. novicida*, the operon for *carAB* has not been formally experimentally defined. Qin and Mann (2006) from their work in Schu S4 proposed that the *carAB* operon in *Francisella* would include the third gene, *pyrB*, prior to *carAB* in all strains of *Francisella*; thus, we sought to experimentally verify the operon in *F. novicida*. Operon prediction programs including BioCyc and MicrobesOnline predict that only carA and carB are co‐transcribed in an operon, and that pyrB is a separate transcriptional unit, while OperonMapper predicts that all 3 are in one operon (Supplemental Figure S2). An analysis using PATRIC (Wattam et al. 2013) of over 20 *Francisella* genomes revealed that in all cases, *carAB* and *pyrB* genes were colocalized, but the location and orientation of the genes upstream and downstream of these three genes are not conserved in all the strains (Figure 2 and Table S2). For example, the serine transporter gene *sdaC* sits immediately 5′ of *carA* in *F. novicida*, but not in *F. tularensis* Schu S4 or *F. philomiragia* 20517. Similarly, a histidine acid phosphatase gene resides 3′ of *pyrB* in *F. novicida* and *F. holarctica* LVS, but is absent from this locus in Schu S4 and *F. philomiragia* (Table S2).

**Figure 3:**
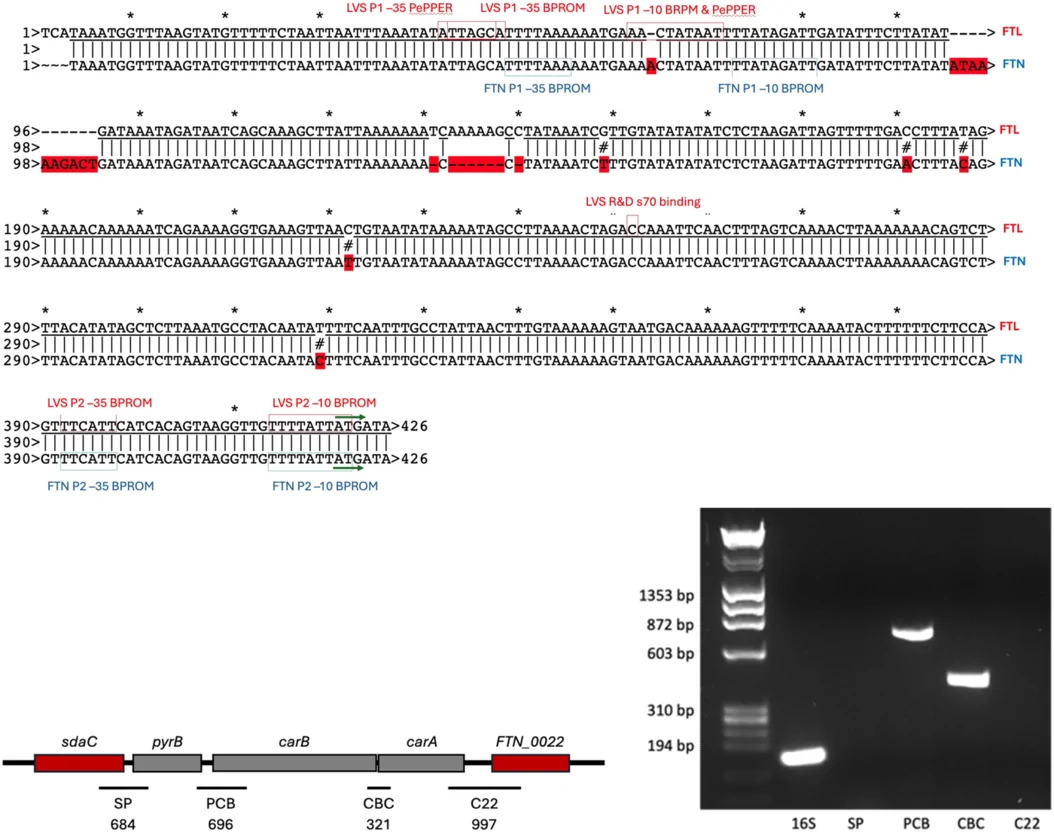
Graphical representation of the *carAB* operon and the RT‐PCR strategy. Alignment of the experimentally determined promoter region of *Francisella tularensis* LVS (NC_007880.1) on top with the putative promoter region of *Francisella novicida* U112 (NC_008601.1) (bottom sequence). Global pairwise alignment was generated in APE (A Plasmid Editor) using the Needleman‐Wunsch algorithm with default match/mismatch and gap parameters. Dashes indicate gap insertions introduced to maximize the alignment score, red highlights denote mismatched positions relative to the *F. tularensis* LVS (FTL) query, and asterisks mark every tenth nucleotide. The putative P1 and P2 of *F. tularensis* LVS are indicated, as predicted by ProPr v2.0 and BPROM. The experimentally determined σ^70^ binding site in *F. tularensis* LVS from Ramsey et al. (2015) is also shown (top sequence). The BPROM predicted putative *F. novicida* P1 and P2 sites are shown in the bottom sequence. The rectangles (gray) represent the *carABpyrB* operon, and the red rectangles show the neighboring genes. The lines/labels below the rectangles represent the sizes and approximate location of the primer sets used in PCR analysis. The lanes of the agarose gel electrophoresis result are labeled with the primer set name that was used. All forward and reverse primers were gene‐specific and are listed in Table S3. PCR products are from the first‐strand cDNA. Observed band sizes correspond to predicted sizes. Representative image (*n* = 3).

To determine if *carA*, *carB*, and *pyrB* are co‐transcribed, we performed RT‐PCR on mRNA with PCR primers spanning the regions between the genes, from *carA* to *pyrB*, following the models of studies for determining *fig* and *capB* operons in *Francisella* (Su et al. 2007; Kiss et al. 2008; Ma et al. 2019). Primer sets (Table S3) were used to amplify first‐strand cDNA across intergenic regions: FTN_0022–*carA*, *carA*–*carB*, *carB*–*pyrB*, and *pyrB*–*sdaC*. Primer set 1 (FTN_0022–*carA*) did not yield the expected 997 bp amplicon (Figure 3), indicating that FTN_0022 is not co‐transcribed with *carA*. Primer set 2 (*carA*–*carB*) produced the expected 314 bp amplicon, confirming co‐transcription of *carA* and *carB*. Primer set 3 (*carB*–*pyrB*) yielded the expected 696 bp amplicon, further supporting co‐transcription. Primer set 4 (*pyrB*–s*daC*) did not generate the expected 321 bp amplicon, indicating *pyrB* and *sdaC* are not co‐transcribed. The presence of these amplicons collectively demonstrates that *carA*, *carB*, and *pyrB* are co‐transcribed as part of the *carABpyrB* operon (Figure 3c).

### 3.2 In Silico Identification of the *carA* Promoter Elements in the *carABpyrB* Operon

To predict the potential promoter elements of the operon, we examined the intergenic nucleotide sequence immediately upstream of *carA* all the way to the sequence of the previous gene (FTN_0022), which is transcribed in the opposite direction. In the genome of *F. novicida* U112, the *pyrB* gene is locus FTN_0019, the large CPS subunit *carB* is FTN_0020, and the CPS small subunit *carA* gene is FTN_0021. We demonstrated above that these genes are transcribed in the same direction, as shown in Figures 2 and 3, from *pyrB* to *carB* to *carA*.

We used the Softberry program BPROM for promoter‐element prediction (Table S5) (Solovyev and Salamov 2011). The sequence selected includes the start codon of *carA* (FTN_0021) to the start codon of the prior gene (in the opposite orientation), histidine acid phosphatase (hap, FTN_0022) (CP009633.1:197157‐203650 *F. novicida* U112). When Softberry's BPROM program was used to analyze this sequence, it identified two putative transcriptional regulatory elements. Two putative promoters were identified: P1, a high‐confidence site (LDF score 8.76) located 375 bp upstream of the *carA* start codon, and P2, a lower‐scoring, more proximal site (LDF score 1.77) located 70 bp upstream of the start codon. The distal P1 site exhibits canonical σ^70^ spacing of 17 bp, while the proximal P2 site falls within the relaxed σ^70^ range with a 16 bp spacer. P1 is farther away from the *carA* start site at position 83 of the promoter region, with a −10 box (TTATAGATT) identified at position 73 and a −35 box (TTTAAA) at position 50. The −35/−10 spacing (end of −35 to start of −10) is 17 bp, consistent with canonical σ^70^ architecture. The second site P2 is much closer to the *carA* gene start site, at position 385, with a −10 box (TTTCAAAAT) at position 369, and a −35 box (ATGACA) at position 355 (Table S5). The spacing is 16 bp, which is within the relaxed σ^70^ range. We designated the upstream, higher‐scoring site as P1 and the proximal, lower‐scoring site as P2, mirroring the P1/P2 terminology used for *E. coli carA* (Figure 3) (Bouvier et al. 1984; Ramond et al. 2015).

Ramsey et al. (2015) predicted the σ^70^ binding site in *F. tularensis* LVS for the same operon and demonstrated that the *carA*‐*carB*‐*pyrB* cluster is organized as a single rightward‐transcribed unit beginning with *carA*‐*carB*‐*pyrB* (FTL_0030 → FTL_0029 → FTL_0028). A high‐confidence σ^70^ peak sits ~40 bp upstream of *carA*, and RNA‐seq reads run continuously across all three cistrons, confirming co‐transcription of these three genes in an operon in *F. tularensis* LVS (Ramsey et al. 2015).

### 3.3 Growth Defects in *carA, carB*, and *pyrB* Mutants Are Rescued by Uracil Supplementation

**Figure 4:**
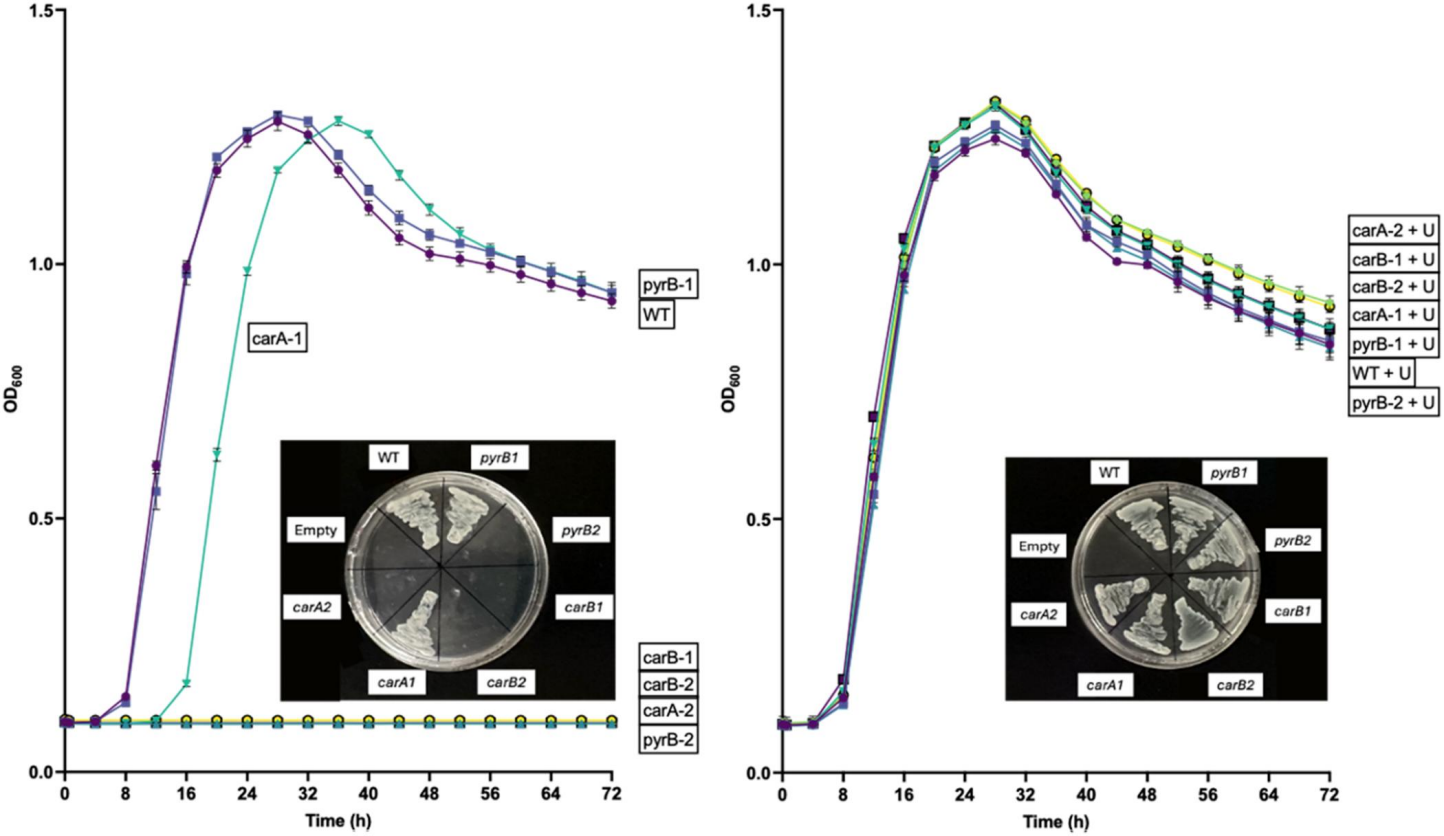
Growth of *Francisella novicida* and *F. novicida carABpyrB* transposon mutants in CDM for 72 h (A) without and (B) with uracil following Maier et al. 2006. Growth of all transposon mutants (with the exceptions of *carA1* and *pyrB1*) shows complete growth deficiency through 72 h. The growth of *pyrB1* appeared similar to that of the wild type, and *carA1* showed a lag phase delay of 12 h. The growth of mutants that we demonstrated were deficient in CDM was restored by the addition of 50 µg/mL uracil. Mutants *carA1* (tnfn1_pw060328p04q165) and *pyrB1* (tnfn1_pw060510p01q109) did not demonstrate the uracil‐rescued phenotype and so were excluded from further study to exclude potential off‐target effects. **Inset Agar Plates**: (A) The CDM agar plates without uracil demonstrate normal growth of wild‐type *F. novicida* (WT), *pyrB1* and *carA1*, as well as the growth defect of *carA2*, *carB1*, *carB2*, and *pyrB2* mutants. (B) These growth defects were reversed with the addition of 50 µg/mL uracil to the CDM agar (Maier et al. 2006).

Following work in LVS by Maier et al. (2006) who showed uracil auxotrophy for *pyrD* and *carA*, we next sought to test the effect of *carA, carB*, and *pyrB* mutants on in vitro growth in *F. novicida*. Our first hypothesis was that strains that are defective for carbamoyl synthesis would show significant defects in growth in CDM. We noticed that in rich medium (TSB‐C), mutants in the pyrimidine biosynthetic pathway reached only about two‐thirds of the wild‐type growth level (data not shown). To more precisely assess these growth defects, cultures of wild‐type *F. novicida* and transposon mutants (*pyrB1::Tn5kan*, *pyrB2::Tn5kan*, *carB1::Tn5kan*, *carB2::Tn5kan*, *carA1::Tn5kan*, and *carA2::Tn5kan*; full mutant designations listed in Table S1) were grown overnight in CDM, a chemically defined minimal medium (Chamberlain 1965) and then plated on CDM agar. We ovserved that four of the mutants (*pyrB2*, *carA2*, *carB1*, and *carB2*) were growth‐deficient on CDM agar (Figure 4A *insets*). We also performed growth studies in CDM broth over 72 h. The growth studies in CDM broth revealed a complete growth inhibition in transposon mutants of *pyrB2*, *carA2*, *carB1*, and *carB2*. The *carA1* transposon mutant showed a growth delay of 10 h in CDM compared to wild type (Figure 4A). We showed that CDM agar supplemented with 50 µg/mL uracil restored the growth deficiency of these mutants on agar and in broth (Figure 4B), as predicted. The growth defect caused by impaired pyrimidine synthesis should be rescued by the addition of exogenous uracil, which enables restoration of pyrimidine nucleotide production through the salvage pathway (bypassing *pyrF*). This was demonstrated by Maier et al. (2006) for the *ΔcarA* mutant in *F. tularensis* LVS. In Figure 4, the growth of *pyrB1* and *carA1* transposon mutants was NOT rescued by uracil, suggesting that these mutants may have other growth‐inhibiting effects due to their transposon insertions unrelated to pyrimidine metabolism. Given this lack of uracil‐rescue, strains *pyrB1* (tnfn1_pw060510p01q109) and *carA1* (tnfn1_pw060328p04q165) were excluded from further study to exclude off‐target effects. This result confirms another prior metabolic study of the same *F. novicida* transposon mutants, in which uracil supplementation was shown (Enstrom et al. 2012). In addition, as expected, arginine supplementation (50 µg/mL) did not rescue the growth phenotype in the *carA2 or carB mutants*, as wild‐type *F. novicida* is naturally auxotrophic for arginine (Ramond et al. 2015). While full growth studies were not performed on *pyrC*‐*pyrF* mutants, as they were not part of the operon characterization, we show growth and uracil rescue data in MMHB in Figure S3.

These findings support the model that strains defective for early steps of pyrimidine nucleotide biosynthesis show defects in growth in CDM media. Similar growth inhibition due to pyrimidine pathway mutations has been reported in *Staphylococcus aureus* (DiMaggio et al. 2025) and *E. coli* (Charlier et al. 2018), among many other bacteria.

### 3.4 Biofilm Assay in MMHB Reveals Biofilm Regulation by Pyrimidine Biosynthesis Genes

We first determined the levels of biofilm measured at 48 h after growth of these bacteria in MMHB media, a limited bacterial growth media previously demonstrated to support limited biofilm production for *F. novicida* (Margolis et al. 2010). In that study, Margolis et al. (2010) did not find a significant effect of *carABpyrB* mutants on biofilm formation, but their experimental design did not account for mutants showing reduced growth. However, Margolis et al. did show that mutation in one gene in the neighborhood of the *carABpyrB* operon, FTN_0031, which encodes a LysR family transcriptional regulator, inhibited biofilm formation. We were able to recapitulate the biofilm defect in the FTN_0031 mutant in this study. We reassessed all the pyrimidine biosynthesis mutants for biofilm and growth, and we report the biofilm:growth ratios in Table S4.

To identify regulators of biofilm production under nutrient stress, we analyzed biofilm levels of *F. novicida* transposon mutants grown in MMHB which showed an auxotrophic phenotype when grown in CDM or MMHB (Figures 4 and S3).

Notably, several downstream pyrimidine metabolism genes such as *pyrG* (CTP synthase), *pyrH* (UMP kinase), *thyA* (thymidylate synthase), *tmk* (dTMP kinase), and *cmk* (CMP kinase) had no transposon insertion mutants available in the library (Gallagher et al. 2007). These genes are essential for *Francisella* viability (Gallagher et al. 2007; Ireland et al. 2019). Thus, these genes could not be evaluated for their biofilm phenotypes. The essentiality of these genes underscores the centrality of nucleotide metabolism to *Francisella* growth, while the presence of viable mutants in *carA*, *carB, pyrB, pyrE*, and *pyrF* allowed identification of biofilm regulation points further upstream in the pathway, where uracil supplementation was able to restore both growth and biofilm phenotypes, even in *pyrE or pyrF* mutants.

**Figure 5:**
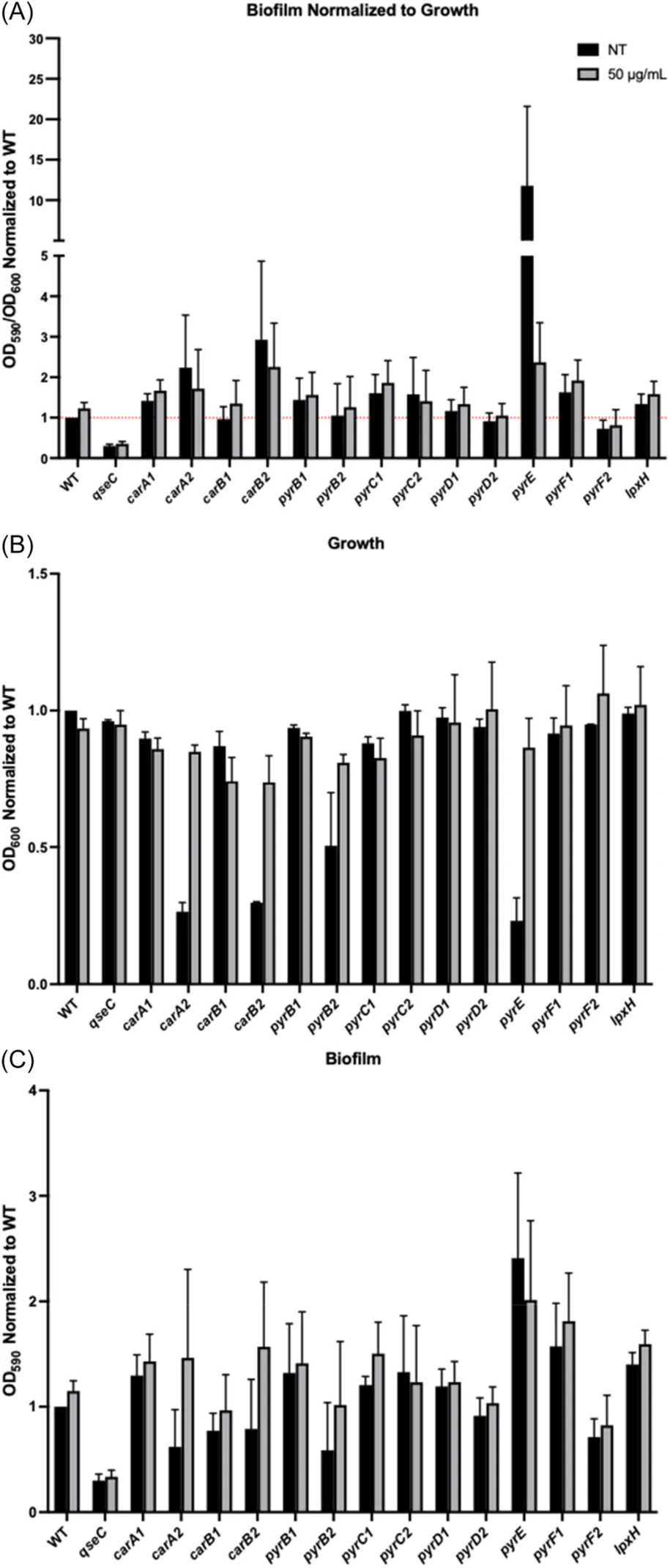
Growth and biofilm formation in *Francisella novicida* pyrimidine pathway mutants in modified Mueller–Hinton broth (MMHB). Biofilm and growth phenotypes of the *qseC* biofilm‐deficient control and transposon mutants in the *carABpyrBCDEF* pathway were measured after growth in MMHB with or without 50 µg/mL uracil. Values were normalized to the untreated wild type, set to 1.0. (A) Biofilm:growth ratios calculated from crystal violet staining normalized to OD_600_. The untreated *pyrE* mutant showed an 11.8‐fold increase relative to wild type. (B) Growth measured by OD_600_. c*arA‐2*, *carB‐2*, *pyrB‐2*, and *pyrE* showed significant growth defects in MMHB, which were restored by uracil supplementation. (C) Biofilm measured by crystal violet staining at OD_590_. The *pyrE* mutant showed increased biofilm formation without normalizing for growth (*p* = 0.0016). Statistical significance was determined by two‐way ANOVA with Dunnett's multiple comparisons test using untreated wild type as the reference control.

To characterize the regulation of biofilm production under pyrimidine stress, we measured the extent of biofilm formation of the wild‐type *F. novicida* and the *carABpyrBCDEF* transposon mutants using a crystal violet 96‐well assay as we have previously described (Durham‐Colleran et al. 2010; Chung et al. 2014; Dean et al. 2015) in modified Mueller‐Hinton Broth (MMHB) (Figure 5). We calculate the Biofilm:Growth ratio (Figure 5A) by dividing the total biofilm crystal violet measurement (Figure 5C) by the growth measurement (Figure 5B), which results in a growth‐normalized biofilm measurment. Using this BF:G ratio enables us to identify the effect of mutants on the biofilm formation that are not due to significant growth effects. For instance, we found that the *carA2*, *carB2*, *pyrB2*, and *pyrE* transposon mutants were significantly growth‐inhibited compared to wild‐type (*carA2*, *carB2*, and *pyrB2* dropped 3.3‐fold, *p* < 0.0001; and *pyrE* dropped 6.8‐fold, *p* < 0.0001; two‐way ANOVA; strain: F(15,64) = 24.40, *p* < 0.0001; uracil: F(1,64) = 39.33, *p* < 0.0001; interaction: F(15,64) = 12.03, *p* < 0.0001; Figure 5B).

Despite the growth inhibition of *pyrE* compared to wild‐type, we found that the *pyrE* transposon mutant produced abundant biofilm (3.3‐fold increase compared to WT, *p* = 0.0016). When normalized for growth, we found the *pyrE* transposon mutant had a BF:G ratio of 11.8‐fold more than WT (adjusted *p* < 0.0001; Figures 5A,C). Interestingly, within the *carABpyrB* operon mutants, *carB2* displayed a notable upward trend in normalized biofilm formation (reaching nearly threefold of WT levels) that partially receded upon uracil supplementation, while *carA2* showed a marginal increase and *pyrB2* remained at baseline; however, none of these achieved statistical significance (Table S4).

We showed that the addition of 50 µg/mL uracil restored the growth deficiency of all these growth‐deficient mutants on agar and in broth (in agreement with Maier et al. 2006), and with this growth restoration, the biofilm production of the *pyrE* transposon mutant was returned to baseline. Thus, uracil rescues both the growth and the biofilm phenotype of the *pyrE* mutant. Due to the fact that there is only one *pyrE* transposon mutant in the library, we were unable to test additional mutants of this gene.

We noted that this biofilm phenotype in MMHB was more exaggerated in the *pyrE* transposon mutant than when grown in tyrpic‐soy broth with 0.1% cysteine (TSB‐C, 11.8‐fold increase vs. 2.23‐fold increase; Figure S4), suggesting that pyrimidine limitation under pyrimidine stress of the MMHB media more strongly activates biofilm production through *pyrE*‐related steps, i.e., the depletion of extracellular pyrimidines. As cultures approach the stationary phase, the salvage pathway flow‐through may be reduced, and the bacteria might compensate by upregulating de novo pyrimidine synthesis genes.

As a control for a known effect on *F. novicida* biofilm formation, the *qseC* sensor histidine kinase mutant (FTN_1617), which we previously characterized, displayed a dramatic reduction in biofilm formation but not growth, in agreement with our earlier findings (Durham‐Colleran et al. 2010; Dean and van Hoek 2015). In further agreement with our results, Ahlund et al. (2010) previously showed almost no impact of *F. novicida carABpyrB*, *pyrD*, or *pyrF* mutants on bacterial growth in TSB‐C media, a media‐specific effect which we also observe.

Together, these data reveal the importance of the pyrimidine biosynthesis pathway on the growth and biofilm formation in *F. novicida* (Durham‐Colleran et al. 2010; Chung et al. 2014; Dean et al. 2015).

### 3.5 Uracil Supplementation Modulates Transcription of the *carABpyrB* Operon

**Figure 6:**
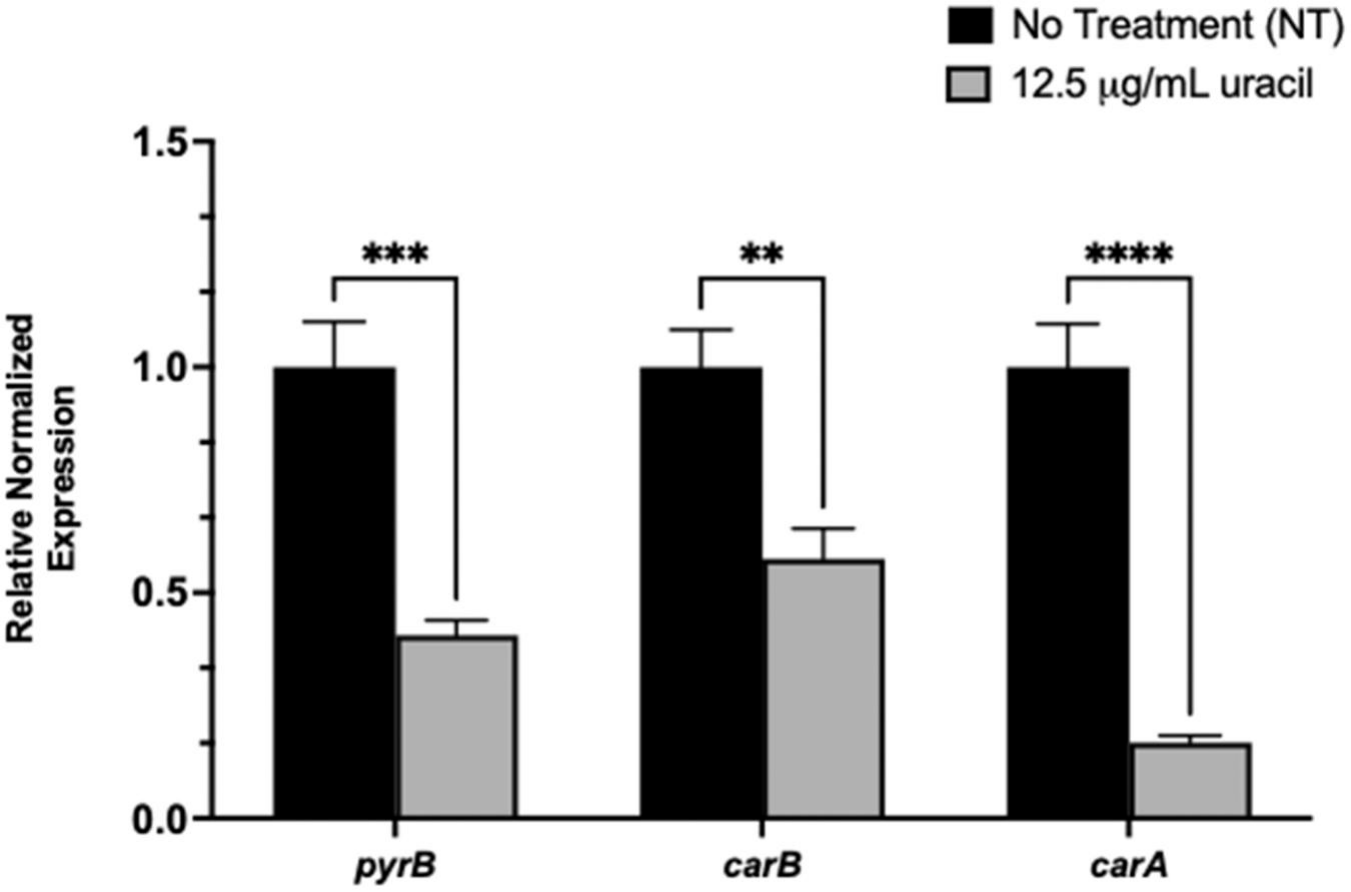
Effect of uracil supplementation on *carA*, *carB*, and *pyrB* gene expression in Chamberlain's Defined Medium (CDM). *Francisella novicida* was grown in CDM with or without uracil (12.5 µg/mL). Cells were collected after 12 h of growth, and RNA was extracted using TRIzol. Relative quantification of gene expression was normalized to 16S rRNA. Conclusion: These results suggest that uracil inhibits transcription of the *carABpyrB* operon, regulating the expression of *carA*, *carB*, and *pyrB*. Statistical significance was determined by two‐way ANOVA; **p = 0.0043; ***p = 0.0003; ****p < 0.0001.

Disruption of certain *F*. *novicida* pyrimidine biosynthesis genes altered their growth and biofilm formation compared to the wild type U112 strain. To confirm that this phenotype could be linked to changes in pyrimidine metabolism via gene expression, we measured transcription of the *carABpyrB* operon under varying uracil conditions. When *F. novicida* was grown in CDM supplemented with 12.5 µg/mL uracil, transcript levels of *pyrB*, *carB*, and *carA* decreased (approximately 2.2‐fold, 1.5‐fold, and 6.5‐fold, respectively), relative to non‐supplemented controls (Figure 6A). This transcriptional downregulation in response to exogenous uracil supplementation supports the presence of feedback regulation within the pyrimidine biosynthetic pathway.

### 3.6 Possible Polar Effect in *pyrE* Mutant

**Figure 7:**
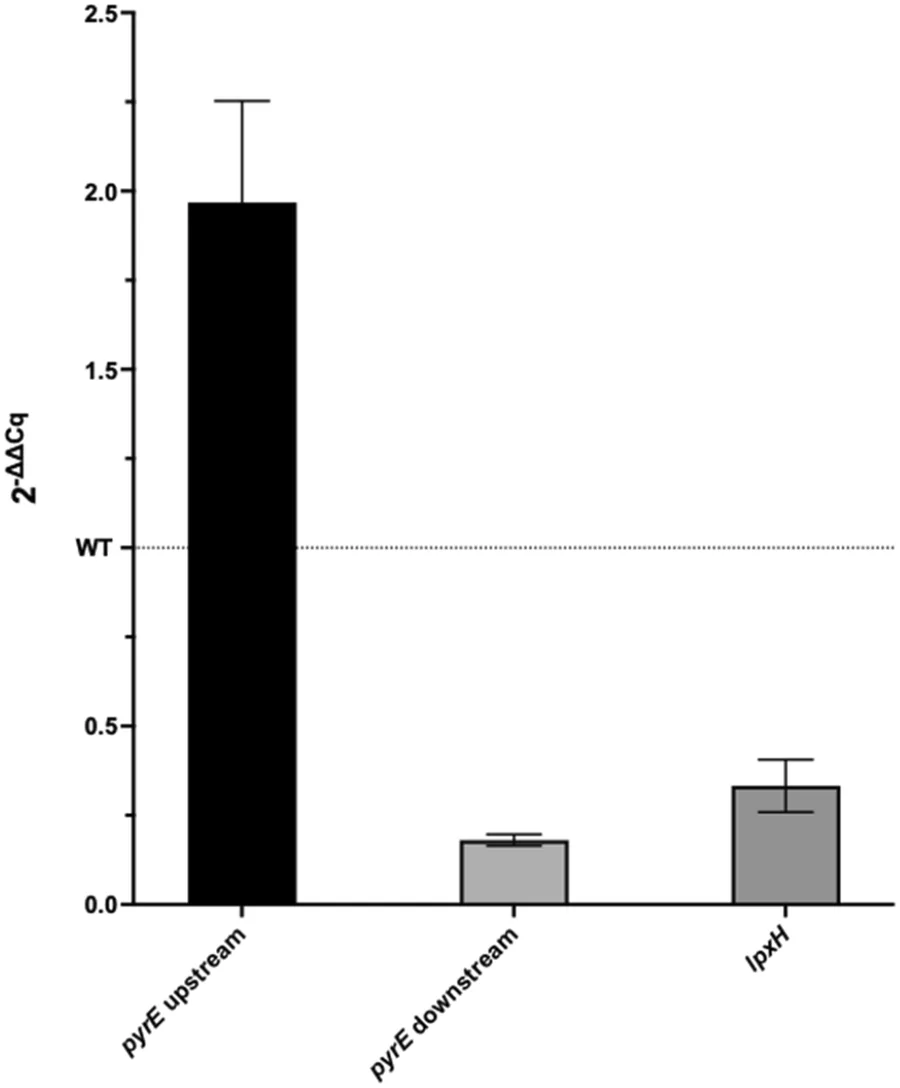
Transcription of *pyrE* (upstream and downstream of transposon insertion) and *lpxH* in WT and *pyrE* transposon mutant. Compared to WT, the *pyrE* transposon mutant shows an increase in the transcription of the region upstream of the transposon insert, but a significant decrease in transcription of the downstream portion of *pyrE*. Transcription of *lpxH* is also decreased in the *pyrE* mutant relative to WT.

When we examined the additional pyrimidine pathway mutants whose growth defects were rescued by uracil, we found that the *pyrE* mutant grew poorly but produced elevated levels of biofilm, resulting in an increased biofilm:growth ratio of approximately 3‐fold in TSB‐C (Figure S4) and 11.8‐fold in MMHB (Figure 5A). The MMHB growth deficiency was rescued by uracil supplementation (Figure 5B), while the MMHB biofilm phenotype was mostly but not completely reduced by uracil (still ~2x), as shown by both raw biofilm and growth‐normalized biofilm measurements (Figures 5A,C, respectively). Because *pyrE* is adjacent to the LPS biosynthesis gene *lpxH*, we next examined whether the transposon insertion in *pyrE* produced a polar effect on *lpxH* expression that may contribute to the partial uracil‐resistant biofilm phenotype seen in *pyrE*. Accordingly, we conducted qPCR on WT and *pyrE* transposon mutants (Figure 7) and found that *pyrE* transcription upstream of the transposon insertion site in the *pyrE* transposon mutant was increased relative to WT (*p* = 0.77, unpaired T‐test of ΔC_T_ value). In contrast, transcription downstream of the insertion site was markedly reduced to ~0.25‐fold of WT (*p* ≤ 0.0001, unpaired T‐test of ΔC_T_ value). Expression of *lpxH*, located downstream of *pyrE*, was reduced to ~0.4‐fold relative to WT (*p* = 0.0035, unpaired T‐test of ΔC_T_ value). The transposon insertion in *pyrE* was associated with reduced downstream *pyrE* transcription and reduced *lpxH* expression, consistent with a polar effect. The increase in upstream transcript abundance may reflect transcriptional accumulation due to premature termination or altered transcript stability upstream of the insertion site. When tested, the *lpxH* transposon mutant did not produce significantly increased levels of biofilm compared to wild type, nor did supplementation of growth medium with uracil impact its growth or biofilm measurements for the *lp*x*H* mutant (Figure 5). However, the transposon insertion inthe one available *lpxH* mutant is very late in the gene, thus this likely does not represent a loss‐of‐function mutant for this gene. Thus, we can not separate the hyper‐biofilm phenotype associated with *pyrE* disruption from a polar effect on *lpxH* expression.

### 3.7 Effect of Different Media on *F. novicida* and the Impact of *carABpyrB* Mutants

When grown in CDM, the most minimal medium that supports *Francisella* growth, *carA*, *carB*, and *pyrB* mutants displayed clear uracil auxotrophy, consistent with strong dependence on de novo pyrimidine biosynthesis under defined nutrient conditions (Figure 4). In MMHB, a nutrient‐limited medium, these mutants also showed reduced growth but also showed elevated biofilm ratios, indicating that disruption of the *carABpyrB* operon alters biofilm regulation under nutrient stress (Figure 5 and Table S4). In contrast, phenotypes were less pronounced in rich TSB‐C medium (Figure S4), where salvageable pyrimidines or related metabolites in the media may partially compensate for loss of de novo biosynthesis via the salvage pathway. These media‐dependent phenotypes suggest that pyrimidine availability influences both growth and biofilm formation in *F. novicida*.

## 4 Discussion

Nutrient‐sensing mechanisms, small molecule communications, glycosyl hydrolases, and iron acquisition systems have all been demonstrated to impact biofilm formation in *Francisella* (Durham‐Colleran et al. 2010; van Hoek 2013; Dean and van Hoek 2015; van Hoek et al. 2019; Chung et al. 2014; Dean et al. 2015, 2020; Verhoeven et al. 2010; Margolis et al. 2010; Biot et al. 2020; Champion et al. 2019; Mlynek et al. 2021, 2024; Siebert et al. 2019, 2020; Soto et al. 2015; Zogaj et al. 2012, Dean et al. 2009). Since *F. novicida* persists in natural aquatic reservoirs in the environment, its ability to form and disperse biofilms relies on a complex interplay of environmental and metabolic signals. Further clarifying the genetic and biochemical foundations of this regulation will improve our understanding of environmental adaptations in *Francisella*.

One goal of the current study was to demonstrate the “*carAB* operon“ structure and characterize pyrimidine biosynthesis regulatory mechanisms in *F. novicida* U112, including predicting the promoter and confirming the operon. We characterized the full‐length transcript of the “*carAB* operon” in *F. novicida* U112. Our data showed that three genes, *carA*, *carB*, and *pyrB*, comprise the *carABpyrB* operon, confirming the suggestion in Schu S4 by Qin and Mann (2006) and in agreement with Ramsey et al. (2015) in LVS.

Through promoter prediction analyses, we identified putative ribosomal binding sites, suggestive of two promoters within the *carA* promoter region, similar to what was reported for the *E. coli carA* promoter (Bouvier et al. 1984; Charlier et al. 2018). Unlike in *E. coli*, where the P1 promoter is regulated by the pyrimidine availability and P2 is regulated by arginine, via *ArgR*, there is no *ArgR* transcriptional regulator identified in the genome of *F. novicida*, nor in *F. tularensis* nor the live vaccine strain (LVS). Other environmental members of the *Francisella* genus do have *ArgR*. In many organisms, arginine is an output of the *carAB* metabolism and thus is a negative regulator of the pathway via *ArgR* in *E. coli*. However, the *argR* gene is not present in *F. novicida*, which is also auxotrophic for arginine and does not carry out de novo arginine synthesis, acquiring it by uptake from the host or the environment (Maier et al. 2006; Ramond et al. 2015). Maier et al. (2006) identified arginine decarboxylase *speA* (FTT_0432) as being important for growth of *F. tularensis* LVS on CDM, a gene which is absent in the *F. novicida* U112 genome, perhaps explaining the arginine auxotrophy in *F. novicida* and further differentiating it from other *Francisella* species with more complete arginine biosynthetic capabilities. Another transcriptional regulator may be able to participate in the regulation of expression via the P2 promoter of this locus in *Francisella*.

The disruption of *carA*, *carB*, and *pyrB* through transposon mutagenesis resulted in growth deficiencies in nutrient‐limited environments that could be rescued with the addition of uracil (*carA2, carB1/2, pyrB2*), but not by arginine, demonstrating that their defect was in the pyrimidine biosynthesis pathway. Our findings with in vitro uracil rescue of *carABpyrB* mutants in *F. novicida* are similar to prior findings in *Francisella* (Maier et al. 2006; Enstrom et al. 2012), further supporting the role of the *carABpyrB* operon and the pyrimidine biosynthesis pathway in the genus *Francisella*. These results are also similar to the results in other bacterial species, such as *Xanthomonas* and *Pseudomonas*, where in *Xanthomonas citri*, the *carB* gene most affected biofilm production (Zhuo et al. 2015), and in *Pseudomonas*, it was the *carA* gene (Butcher et al. 2016).

We measured for changes to *F. novicida* biofilm formation in MMHB due to additional pyrimidine biosynthesis gene mutants outside of the *carABpyrB* operon (transposon mutants of *pyrC‐F*) (Figure 5). In contrast to our earlier observations in rich media (TSB‐C), where mild increases were noted (Figure S4), our MMHB data reveal that only the *pyrE* mutant displays a large, statistically significant increase in biofilm. Strains disrupted in *pyrC*, *pyrD*, and *pyrF* showed no significant effects in normalized biofilm production under these conditions, remaining at wild‐type baseline. Within the *carABpyrB* operon mutants, *carA2* and *carB2* trended upward at approximately twofold and threefold of WT levels, respectively, but these variations did not meet statistical significance. The data can also be understood in temporal terms in the bacterial growth phases, from log phase (rapid planktonic growth) to the stationary phase which supports biofilm production. In rich media or the early log phase, the salvage pathway is favored. So, the model is one of growth‐phase‐conditional partitioning where the salvage pathway dominates during early/rapid growth in rich media. As nutrients (and pyrimidine precursors) are consumed and cultures enter the stationary phase or when media are minimal, the network flips its requirements, and de novo pyrimidine biosynthesis is emphasized.

We demonstrated that different media can significantly impact the effect of the pyrimidine mutants on biofilm production. De novo pyrimidine synthesis is likely required during growth in more minimal media (MMHB, CDM), while the salvage pathway is likely utilized in rich media (TSB‐C), supported by our findings that uracil addback to MMHB normalizes biofilm:growth ratios while CDM highlights the uracil dependence. This model is further supported by the results of Holland et al. (2017), who demonstrated that the protein expression of PyrC (FTL_0033) is significantly higher during *F. tularensis* LVS growth in MHB compared to Brain Heart Infusion pH 6.8 (BHI, a rich medium) (Hazlett et al. 2008) (BHI/MHB = 0.657, *p* = 0.035). This supports the concept that de novo pyrimidine synthesis is likely required during growth in the more limited media. In addition, CarA (FTL_0030), CarB (FTL_0029), and PyrB (FTL_0028), as well as PyrD (FTL_0046), PyrE (FTL_0507), and PyrF (FTL_0045) protein expressions were also higher in MHB, but the changes were not statistically significant in the Holland et al. study. In contrast, Upp (UPRT) protein levels were shown to be almost 2.5‐fold higher during *F. tularensis* LVS growth in BHI (rich media) compared to MHB (BHI/MHB ≈ 2.44; trend, *p* ≈ 0.14), representing the importance of the salvage pathway in rich media (Holland et al. 2017). That is the expected outcome when exogenous nucleobases and nucleosides are abundant as in BHI.

As a culture undergoes entry into the stationary phase or undergoes nutrient depletion, this leads to favoring de novo pyrimidine synthesis. This concept is supported by the iTraq proteomics results of Lenco et al. (2009) (shown in Holland et al. 2017, “stationary phase vs. log phase”), demonstrating that *F. tularensis* Schu S4 bacteria in the stationary phase (potentially able to form biofilms) had significantly more CarA, CarB, and PyrB protein than during the rapidly dividing exponential phase of growth, which would not be forming biofilm. PyrB, CarB, and CarA proteins were more abundant in the stationary phase than in the exponential phase when grown in CDM (CarA [2.20–2.27×, *p* < 0.001]; CarB [1.88–1.98×, *p* < 0.001]; PyrB [2.39–2.52×, *p* ≤ 0.008]; PyrC [1.62–1.83×, *p* ≤ 0.013]). Other proteins in the pyrimidine pathway were also elevated in the stationary phase, but by less than the twofold cutoff (PyrF = 1.84 > PyrC > PyrE > PyrD > PyrH = 1.08) (Lenco et al. 2009). This represents a growth phase switch toward de novo pyrimidine synthesis capacity when external pools of pyrimidines are spent. This model provides mechanistic coherence with our experimental observations. The biofilm phenotypes that we observed match this switch. In MMHB, the biofilm phenotypes are exaggerated, precisely when cells rely on de novo pyrimidine synthesis. In TSB‐C + uracil, the salvage pathway is available, which rescues growth and normalizes biofilm (BF:G).

Depletion of extracellular pyrimidines as cultures approach the stationary phase lowers the salvage flux; cells compensate by upregulating *carABpyrB and pyrCDEF*. Published data demonstrating the binding of the σ^70^ to the *carA* promoter (Ramsey et al. 2015) and OxyR binding (via ChIPseq) at *carA* (Al Ahmar et al. 2019) is compatible with stationary phase responsive upshift of de novo transcription. So, our model is one of growth‐phase‐ conditional partitioning. In this model, the salvage pathway dominates during early/rapid growth in rich media. As nutrients (and pyrimidine precursors) are consumed and cultures enter the stationary phase or when media are minimal, the network flips its requirements, and de novo pyrimidine biosynthesis is emphasized. Minimal media predispose the stationary‐like state for this pathway.

### 4.1 *pyrE* (FTN_0529)

We observed a large effect on biofilm for the mutants of *pyrE* (FTN_0529). We found that the single *pyrE* mutant showed significantly decreased growth with increased biofilm compared to the wild type U112 bacteria, even when not accounting for growth. When biofilm measurements for *pyrE* mutants were normalized to growth and compared to WT biofilm measurements also normalized to growth, there was a robust and statistically significant increase in biofilm produced by the *pyrE* mutants. The strong *pyrE* mutant phenotype (with uracil rescue) may be consistent with a metabolic flux chokepoint becoming limiting exactly when the salvage pathway cannot keep up. Interestingly, this is the first study that has identified a role for the *pyrE* gene in *Francisella*. While there is polar effect of the *pyrE* mutant on *lpxH* gene expression, the uracil rescue of the *pyrE* biofilm phenotype suggests that most of this effect is due to the pyrimidine pathway modulation.

### 4.2 Biofilm and Signaling Molecules

Disruption of *pyrE* was associated with increased biofilm production despite reduced growth, indicating that the biofilm phenotype is not simply a consequence of increased biomass. Biofilm phenotypes were not uniform across all pyrimidine biosynthesis mutants, suggesting that the response depends on where de novo pyrimidine biosynthesis is interrupted. Mutations in *carA, carB*, or *pyrB* disrupt early steps required for pathway entry and would be expected to broadly reduce pyrimidine synthesis while also limiting growth. By contrast, PyrE protein functions later in the pathway, catalyzing the conversion of orotate to orotidine‐5′‐monophosphate immediately upstream of UMP production. Loss of *pyrE* would therefore be expected to restrict UMP synthesis while permitting continued formation of upstream intermediates, potentially generating a distinct pyrimidine‐limitation state from that caused by early pathway disruption. This pathway‐position effect may explain why *pyrE* produced the strongest biofilm phenotype relative to growth, while *pyrC*, *pyrD*, and *pyrF* did not produce equivalent effects. Although *pyrE* is adjacent to *lpxH*, the biofilm phenotype was not explained solely by altered *lpxH* expression and was rescued by uracil addition, supporting a model in which *pyrE* disruption creates a specific metabolic state that promotes biofilm formation rather than reflecting only polarity or generalized growth inhibition. Together, these findings suggest that pyrimidine pathway mutants may influence biofilm production indirectly by altering intracellular metabolite pools or signaling states that regulate biofilm assembly, maintenance, or dispersal under nutrient‐limited conditions. These findings also place *F. novicida* within a broader framework in which pyrimidine availability can influence bacterial transcriptional regulation and community‐associated phenotypes. In *Pseudomonas aeruginosa*, disruption of de novo pyrimidine biosynthesis altered colony morphology and mucoidy, while uracil supplementation restored intracellular UMP/UTP pools and the mucoid phenotype through pyrimidine salvage. This response was linked to altered RpoN levels and σ‐factor competition with AlgU at the alginate biosynthesis promoter (Al Ahmar et al. 2019). Although σ‐factor competition was not directly tested in the present study, the predicted σ^70^‐associated promoter elements upstream of *carA* (Ramsey et al. 2015), together with uracil‐responsive regulation of the *carABpyrB* operon, suggest that pyrimidine availability may influence biofilm formation through broader transcriptional regulatory mechanisms in *F. novicida*.

Signaling molecules such as c‐di‐GMP and (p)ppGpp play important roles in *F. novicida* biofilm regulation: elevated c‐di‐GMP stimulates biofilm formation, whereas RelA‐dependent (p)ppGpp signaling is linked to reduced biofilm accumulation and biofilm dispersal. (Zogaj et al. 2012; Dean et al. 2009). *Francisella* ppGpp levels could affect biofilm via PigR modulation of the RNA polymerase, as Ramsey et al. (2015) found MglA–SspA–PigR footprints at nearly every σ^70^ promoter. In *E. coli*, the P1 promoter is subject to stringent control by the alarmone ppGpp, leading to changes in *pyrB* and *carAB* transcription (Bouvier et al. 1984; Charlier et al. 2018). We have previously demonstrated that ppGpp is linked to biofilm production in *F. novicida* (Chung et al. 2014; Dean et al. 2015); however, the potential regulation of the *carABpyrB* operon by ppGpp remains to be experimentally determined in *Francisella*.

While c‐di‐GMP promotes biofilm formation in many bacteria, *F. novicida* and *F. tularensis* differ in their utilization of this pathway, with c‐di‐GMP‐mediated regulation being unique to *F. novicida* (Zogaj et al. 2012). Also, (p)ppGpp has been shown to be a regulator of biofilm production in many bacteria, including *Pseudomonas aeruginosa* and in *F. novicida* (Dean et al. 2009). We recently demonstrated that (p)ppGpp levels are increased following the application of exogenous Burkholderia Diffusible Signal Factor (BDSF) (Dean et al. 2015), which leads to increased levels of *relA* expression, (p)ppGpp, and chitinase expression, with a resulting decrease in biofilm (Dean et al. 2015). The ppGpp signal may regulate an early stage in biofilm formation, while pyrimidine biosynthesis may regulate a late stage in biofilm formation. Further research is needed to elucidate the molecular mechanisms by which these modulations of pyrimidine biosynthesis specifically enhance *F. novicida* biofilm formation.

## 5 Conclusion

In conclusion, our study advances the understanding of the structure and function of the *carAB/pyrB* operon in *Francisella* by confirming that it contains the three genes. Our data demonstrate that pyrimidine biosynthesis may have a positive regulatory function on *F. novicida* growth and a negative regulatory function on *F. novicida* biofilm formation, suggesting a potential link between nucleotide metabolism and environmental adaptation. Such insights may contribute to the development of strategies to control *Francisella* persistence in the environment by targeting biofilm‐related pathways. Ultimately, this work deepens our understanding of *Francisella* environmental survival by revealing that de novo pyrimidine biosynthesis functions as a responsive metabolic control point that can shift the bacteria between growth and biofilm state under nutrient limitation.

## Author Contributions

**Katie C. Sprinkel:** conceptualization, methodology, investigation, data curation, formal analysis, original draft writing and preparation, final review. **Annika R. Geiger:** investigation, data curation, formal analysis, original draft writing and preparation, final review. **Monique L. van Hoek:** conceptualization, methodology, supervision, financial support, data curation, formal analysis, original draft writing and preparation, final review.

## Funding

The authors have nothing to report.

## Ethics Statement

The authors have nothing to report.

## Conflicts of Interest

The authors declare no conflicts of interest.

## Supporting information


**Figure S1:** *Francisella novicida U112* pyrimidine gene neighborhoods from PATRIC (www.bv‐brc.org). The genome shown is NC_008601. A. The genes of focus in this study, *carA, carB* and *pyrB* and neighboring genes are shown, including sdaC (FTN_0018) and *hap*, histidine acid phosphatase (FTN_0022), the latter which is transcribed in the opposite direction. Our hypothesis is that *carABpyrB* is the functional operon, not including *sdaC* and *hap* in *F. novicida.* B. The gene *pyrC* (FTN_0024) has neighbors *tmpT* (FTN_0023) and an uncharacterized gene (FTN_0025) transcribed in opposite directions and is just 3’ of *carA*. C. The two genes *pyrF* (FTN_0035) and *pyrD* (FTN_0036) may also be in an operon. 5’ to *pyrF* is FTN_0034 which is transcribed in the opposite direction. FTN_0037 is an uncharacterized protein gene 3’ of *pyrD*. D. The genes *pyrE* and *lpxH* may be in an operon together.
**Figure S2:** Operon Prediction program results. A. BioCyc prediction of the operon for *carABpyrB*. BioCyc predicts that only *carA* and *carB* are co‐transcribed in an operon, and that *pyrB* is a separate transcriptional unit. In the figure, gene AW25‐RS00915 (Histidine acid phosphatase, WP_003040818.1) is shown in the opposite direction to *carA.* Thus, the prediction from this analysis is that the transcript will not extend from *carB* to *pyrB*. Our RT‐PCR data demonstrates that the transcript does extend from *carB* to *pyrB*, thus the BioCyc model of the operon is not correct. MicrobesOnline predictor gave a similar result. B. OperonMapper. OperonMapper output predicts that *carABpyrB* will be in a 3 gene operon, and that *sdaC* is outside of this operon.
**Figure S3:** Uracil supplementation returns every *F*. *novicida* pyrimidine mutant to the uracil‐treated wild‐type baseline in TSB‐C. Growth (A), biofilm (B) and biofilm:growth ratio (C) of transposon mutants in the pyrimidine biosynthetic pathway, grown in TSB‐C for 48 h without (black bars) and with (grey bars) 50 ug/ml uracil. All values are normalized to the untreated wild type of the same biological replicate, where untreated WT is equal to 1. Bars represent the mean of three biological replicates (*n* = 3) with standard deviation shown by error bars.
**Figure S4:** Normalized Biofilm:Growth Ratio of *carABpyrBCDEF* Mutants in TSB‐C verus MMHB: All transposon mutants are represented as the biofilm:growth ratio, normalized to the wild type (WT) of their respective baseline growth conditions (i.e. all TSB‐C data presented was normalized to the WT grown in TSB‐C), where WT is equal to 1.
**Table S1:** List of Bacterial Transposon Mutants. The strike‐out line is placed on the two mutants that do not demonstrate the uracil‐dependent phenotype and so were excluded from further study. Mutant identification numbers are from the original publication [33].
**Table S2:** Pyrimidine pathway and neighboring genes in *F. novicida* U112, and their orthologs in *F. tularensis* SchuS4 and *F. tularensis* LVS. Adapted from the published table of 1745 genes (functional or inactivated) identified in *F.t. novicida* U112, and their orthologous counterparts in the genome of *F. tularensis* SchuS4 and *F. tularensis* LVS [56].
**Table S3:** Primers Used in This Study for qRT‐PCR.
**Table S4:** Raw Growth, Biofilm and Biofilm:Growth Ratios in *Francisella* Pyrimidine Pathway Mutants in MMHB.
**Table S5:** BPROM promoter analysis of carA 5'UTR. The *carA* promoter region spanning 425 nucleotides upstream of the *carA* ATG site was analyzed with the BPROM v2.0 software (Softberry Inc.) [42] using default parameters for *Escherichia coli* promoter prediction. (GenBank *Francisella tularensis* subsp. *novicida* U112, CP009633.1:197157‐197581). Predictions with scores above 0.8 were considered potential regulatory regions. Putative P1 and P2 −35/−10 elements were identified at position 88 and 385. In additional analysis, ProPr identified two potential σ^54^ binding sites, AACTATAAT and TACAATACT, shown in dark grey with underscore.

## Data Availability

The data that support the findings of this study are available on request from the corresponding author.
