# Supplementary material for: Characterization of carABpyrB operon and role of pyrE in Francisella novicida biofilm
Source: Microbiologyopen. 2026 Sep 7;15(5):e70355. doi: 10.1002/mbo3.70355 (PMC13548406; doi:10.1002/mbo3.70355)
Supplement: Supplementary file 1 [file MBO3-15-e70355-s001.docx]

**Supplementary Information**:

For: Sprinkel, K. C., A. R. Geiger, and M. L. van Hoek. 2026. “Disruption of Pyrimidine Biosynthesis Reprograms Biofilm Formation in *Francisella novicida* Through Metabolic Feedback.” *MicrobiologyOpen* 0: e70355. <https://doi.org/10.1002/mbo3.70355>

**Index:**

**Supplemental Methods:** pg 2.

**Supplemental Data:** pg 3-11.

**Figure S1:** *Francisella novicida U112* pyrimidine gene neighborhoods from PATRIC**.** pg 3.

**Figure S2:** Operon Prediction program results. pg 4.

**Table S1:** List of Bacterial Transposon Mutants. pg 5.

**Figure S3**: Uracil supplementation returns every pyrimidine mutant to the uracil-treated wild-type baseline. pg 6.

**Table S2:** Pyrimidine pathway and neighboring genes in *F. novicida* U112, and their orthologs in *F. tularensis* SchuS4 and *F. tularensis* LVS. pg 7.

**Table S3:** Primers Used in This Study for qRT-PCR. pg 8.

**Table S4:** Raw Growth, Biofilm and Biofilm:Growth Ratios in *Francisella* Pyrimidine Pathway Mutants in MMHB. pg 9.

**Figure S4:** Normalized Biofilm:Growth Ratio of *carABpyrBCDEF* Mutants in TSB-C versus MMHB. pg 10.

**Table S5:** BPROM promoter analysis of carA 5’UTR. pg 11.

**Supplemental Methods:**

**Computational operon prediction:** Input sequences and locus mapping: Computational operon analyses were performed for the *F. novicida* U112 region containing *carA* (FTN_0021), *carB* (FTN_0020), and *pyrB* (FTN_0019), with the upstream oppositely oriented histidine acid phosphatase (FTN_0022). When tools required organism specific locus tags different from *F. novicida* (FTN) identifiers (e.g., AW25RS00915 in *F. tularensis* LVS annotations or internal IDs produced by OperonMapper), we mapped those IDs to the *F. novicida* orthologs by gene symbol and protein annotation (CarA/CarB/PyrB) and conserved synteny (*sdaC–carA–carB–pyrB*). Reproducibility and visualization: All tools were run with default parameters and no organism specific tuning. Screenshots/tables were exported from the respective web interfaces and redrawn for clarity without altering gene order, strand, or the reported probabilities/distances. Accession, coordinates, and locus tag mappings used for each panel are provided in the figure legend and table footnotes. Our RT-PCR results, described in the main text, test these predictions experimentally.

**BioCyc / MicrobesOnline:** We used BioCyc (Pathway Tools operon/Transcription Unit predictor; default parameters) for the *carA–carB–pyrB* neighborhood prediction and exported the gene neighborhood and predicted Transcription Units (TUs) for the orthologous region (accessed 5-11-2025). BioCyc predicted *carA* and *carB* to be cotranscribed in a single TU and *pyrB* to form a separate TU. The upstream histidine acid phosphatase gene was shown on the opposite strand relative to *carA*. We independently queried MicrobesOnline (default operon prediction settings; accessed 5/11/2025), which returned the same qualitative architecture (*carA–carB* together; *pyrB* separate). These predictions were recorded from the web interfaces without manual curation and used to generate **Supplemental Figure S2-A**.

**OperonMapper:** We submitted the genomic FASTA spanning *sdaC–carA–carB–pyrB* (U112, CP009633.1) to OperonMapper (default settings; accessed 9/10/2025 OperonMapper reports pairwise operonic probabilities, intergenic distances, and final operon calls. The output classified *pyrB–carB–carA* as operonic (example pairs: *pyrB* to *carB*, distance ≈ 142 bp, probability 0.94; *carB* to *carA,* distance ≈ 5 bp, probability 0.97) and placed the upstream amino acid permease (*sdaC*) outside this operon (*sdaC* to *pyrB* labeled noOperon, probability 0.97). The final predicted operon therefore comprised *carABpyrB,* consistent with our experimental co-transcription data; these outputs were used to build **Supplemental Figure S2-B** and the accompanying table.

**Supplemental data:**

**Figure S1:** ***Francisella novicida U112* pyrimidine gene neighborhoods from PATRIC (www.bv-brc.org).** The genome shown is NC_008601. **A.** The genes of focus in this study, *carA, carB* and *pyrB* and neighboring genes are shown, including sdaC (FTN_0018) and *hap*, histidine acid phosphatase (FTN_0022), the latter which is transcribed in the opposite direction. Our hypothesis is that *carABpyrB* is the functional operon, not including *sdaC* and *hap* in *F. novicida.* **B.** The gene *pyrC* (FTN_0024) has neighbors *tmpT* (FTN_0023) and an uncharacterized gene (FTN_0025) transcribed in opposite directions and is just 3’ of *carA*. **C.** The two genes *pyrF* (FTN_0035) and *pyrD* (FTN_0036) may also be in an operon. 5’ to *pyrF* is FTN_0034 which is transcribed in the opposite direction. FTN_0037 is an uncharacterized protein gene 3’ of *pyrD*. **D.** The genes *pyrE* and *lpxH* may be in an operon together.

**Figure S2: Operon Prediction program results.** **A. BioCyc** prediction of the operon for *carABpyrB*. BioCyc predicts that only *carA* and *carB* are co-transcribed in an operon, and that *pyrB* is a separate transcriptional unit. In the figure, gene AW25-RS00915 (Histidine acid phosphatase, WP_003040818.1) is shown in the opposite direction to *carA.* Thus, the prediction from this analysis is that the transcript will not extend from *carB* to *pyrB*. Our RT-PCR data demonstrates that the transcript does extend from *carB* to *pyrB*, thus the BioCyc model of the operon is not correct. MicrobesOnline predictor gave a similar result. **B. OperonMapper.** OperonMapper output predicts that *carABpyrB* will be in a 3 gene operon, and that *sdaC* is outside of this operon.

**A.**

**B.**

**Predicted operonic gene pair**

idGen1	idGen2	COGgene1	GOGgene2	Dist	STRING	CLASS	Probability

FPDOONKH_00001	FPDOONKH_00002	COG0814	COG0540	141	NA.	**noOperon**	0.97

FPDOONKH_00002	FPDOONKH_00003	COG0540	COG0458	142	999	Operon	0.94

FPDOONKH_00003	FPDOONKH_00004	COG0458	COG0505	5	999	Operon	0.97

**Predicted Operon:**

Operon	IdGene	Type	COGgene	PosLeft	postRight	Strand	Function

1

FPDOONKH_00001	CDS	COG0814	14	1237	-	[E] Amino acid permeases

2

FPDOONKH_00002	CDS	COG0540	1378	2304	-	[F] Aspartate carbamoyltransferase, catalytic chain

FPDOONKH_00003	CDS	COG0458	2446	5730	-	[EF] Carbamoylphosphate synthase large subunit (split

gene

FPDOONKH_00004	CDS	COG0505	5735	6901	-	[EF] Carbamoylphosphate synthase small subunit

**Table S1: List of Bacterial Transposon Mutants.** The strike-out line is placed on the two mutants that do not demonstrate the uracil-dependent phenotype and so were excluded from further study. Mutant identification numbers are from the original publication [33].

| **Mutant abbreviation** | **Mutant name** | **Gene** | **Transposon** | **Dir** | **Genome Position** | **Position to ORF** |
| --- | --- | --- | --- | --- | --- | --- |
| *~~pyrB1~~* | ~~tnfn1_pw060510p01q109~~ | ~~FTN_0019~~ | ~~T18~~ | ~~R~~ | ~~17434~~ | ~~542(924)~~ |
| *pyrB2* | tnfn1_pw060323p03q119 | FTN_0019 | T20 | R | 17633 | 343(924) |
| *carB1* | tnfn1_pw060323p08q120 | FTN_0020 | T18 | R | 19649 | 1753(3282) |
| *carB2* | tnfn1_pw060328p01q118 | FTN_0020 | T20 | R | 20482 | 920(3282) |
| *~~carA1~~* | ~~tnfn1_pw060328p04q165~~ | ~~FTN_0021~~ | ~~T20~~ | ~~R~~ | ~~22412~~ | ~~161(1164)~~ |
| *carA2* | tnfn1_pw060510p02q160 | FTN_0021 | T20 | R | 21897 | 676(1164) |
| *pyrC1* | tnfn1_pw060328p06q113 | FTN_0024 | T20 | F | 25426 | 547(1344) |
| *pyrC2* | tnfn1_pw060510p02q166 | FTN_0024 | T20 | R | 25115 | 276(1344) |
| *pyrF1* | tnfn1_pw060323p04q176 | FTN_0035 | <KAN-2> | F | 37525 | 308(642) |
| *pyrF2* | tnfn1_pw060510p01q129 | FTN_0035 | T20 | R | 37341 | 124(642) |
| *pyrD1* | tnfn1_pw060323p03q144 | FTN_0036 | T20 | R | 38143 | 280(738) |
| *pyrD2* | tnfn1_pw060510p04q195 | FTN_0036 | T20 | F | 38354 | 491(738) |
| *pyrE* | tnfn1_pw060510p01q127 | FTN_0529 | T20 | F | 551044 | 313(624) |
|  |  |  |  |  |  |  |

**Figure S3: Uracil supplementation returns every *F. novicida* pyrimidine mutant to the uracil-treated wild-type baseline in TSB-C.** Growth (A), biofilm (B) and biofilm:growth ratio (C) of transposon mutants in the pyrimidine biosynthetic pathway, grown in TSB-C for 48 h without (black bars) and with (grey bars) 50 ug/ml uracil. All values are normalized to the untreated wild type of the same biological replicate, where untreated WT is equal to 1. Bars represent the mean of three biological replicates (n = 3) with standard deviation shown by error bars.

**Table S2: Pyrimidine pathway and neighboring genes in *F. novicida* U112, and their orthologs in *F. tularensis* SchuS4 and *F. tularensis* LVS.** Adapted from the published table of 1745 genes (functional or inactivated) identified in *F. novicida* U112, and their orthologous counterparts in the genome of *F. tularensis* SchuS4 and *F. tularensis* LVS [56].

| Locus Tags for Genes in U112 | Locus Tags of Orthologs in SchuS4 | Locus Tags of Orthologs in LVS | Gene Location in U112 Genome | | Predicted Protein Size (aa) | Gene Name | Gene Description | Functional Category and Subcategory | Predicted Protein Localization |
| --- | --- | --- | --- | --- | --- | --- | --- | --- | --- |
| FTN_0017 | Schu  no seq | LVS  no seq | 15027 | 13789 | 413 |  | phage integrase | phage or plasmid related proteins | unknown |
| FTN_0018 | FTT_1668 | FTL_0024 | 16908 | 15688 | 407 | *sdaC* | serine permease | transport - amino acid | unknown |
| FTN_0019 | FTT_1665 | FTL_0028 | 17975 | 17052 | 308 | *pyrB* | aspartate carbamoyl transferase | nucleotides and nucleosides metabolism | unknown |
| FTN_0020 | FTT_1664 | FTL_0029 | 21401 | 18120 | 1094 | *carB* | carbamoyl-phosphate synthase large chain | nucleotides and nucleosides metabolism | unknown |
| FTN_0021 | FTT_1663 | FTL_0030 | 22572 | 21409 | 388 | *carA* | carbamoyl-phosphate synthase small chain | nucleotides and nucleosides metabolism | unknown |
| FTN_0022 | (FTT_1662c) | FTL_0031 | 22992 | 24044 | 351 |  | histidine aid phosphatase | putative enzymes | unknown |
| FTN_0023 | FTT_1661 | FTL_0032 | 24748 | 24071 | 226 | *tmpT* | thiopurine S-methyl transferase | putative enzymes | cytoplasm |
| FTN_0024 | FTT_1660c | FTL_0033 | 24880 | 26223 | 448 | *pyrC* | dihydroorotase | nucleotides and nucleosides metabolism | unknown |
| FTN_0025 | Schu  no seq | LVS  no seq | 26797 | 26213 | 195 |  | hypothetical protein | hypothetical - novel | cytoplasm |
| FTN_0035 | FTT_1648c | FTL_0045 | 37218 | 37859 | 214 | *pyrF* | orotidine 5'-phosphate decarboxylase | nucleotides and nucleosides metabolism | unknown |
| FTN_0036 | FTT_1647c | FTL_0046 | 37864 | 38601 | 246 | *pyrD* | dihydroorotate oxidase | nucleotides and nucleosides metabolism | unknown |
| FTN_0529 | FTT_0437c | FTL_0507 | 551356 | 550733 | 208 | *pyrE* | orotate phosphoribosyl transferase | nucleotides and nucleosides metabolism | unknown |

**Table S3: Primers Used in This Study for qRT-PCR.**

| Primer Set | Dir. | Sequence (5'-3') | Description |
| --- | --- | --- | --- |
| PS1 | F | AGCACTAACCACTGTACGATTT | Amplifies a 997 bp fragment containing partial carA |
|  | R | TCACGCACGATTAGACCTTTAG | and FTN_0022 if these genes were co-transcribed |
| PS2 | F | CGGTACAGTATCACCCAGAAAG | Amplifies a 400 bp fragment containing partial carA and |
|  | R | CACAGTTAAGCGCAGTTTGTC | partial carB if these genes were co-transcribed |
| PS3 | F | AAGCTATCGAAGCAGGTGAAG | Amplifies a a 600 bp fragment containing partial carB |
|  | R | CACTATGTGGTTTAGGGTGAC | and partial pyrB if these genes were co-transcribed |
| PS4 | F | TAACTCGCGTCCAGAGAGAA | Amplifies a 684 bp fragment containing partial FTN_0018 and pyrB if these genes were co-transcribed |
|  | R | ACCAGTAGTATTGGCAAGATAGC |  |
| PS5 | F | AATGAACACCCTACCCAATCTC | Amplifies within pyrB gene for qPCR of pyrB transcription |
|  | R | GAGTGAACCGTACGACCATATT |  |
| PS6 | F | ATGGTGGTGAGCGTGTATTT | Amplifies within carB gene for qPCR of carB transcription |
|  | R | GATAGCGAACGAGTTGGGATAG |  |
| PS7 | F | ATCGTGCGTGAGAACCTTAC | Amplifies within carA gene for qPCT of carA transcription |
|  | R | CTCTTACCGCACCCTCTTTAC |  |
| PS8 | 16SF | CTGTCGTCAGCTCGTGTTGT | Amplifies 156 bp within 16S rRNA; |
|  | 16SR | CGTAAGGGCCATGATGACTT | used as endogenous control |
| polA | F | CGAGGCTGATGATGTAATAGG | Endogenous control for geometric means normalization |
|  | R | CTGTAACTAGCTGAGCCATATC |  |
| pyrE(U) | F | ACGCTTAAGTCAGGACGTATTAG | Amplifies a 124 bp sequence upstream of transposon insertion in *pyrE* |
|  | R | CGTACTTGACATCGCATTTGATAA |  |
| pyrE (D) | F | TGCTGAAATAGCTGGTGTTGTA | Amplifies a 111 bp sequence downstream of transposon insertion *pyrE* |
| pyrE (D) | R | GCTAGAACAGGAATATTGAAATCTTGAG |  |
| lpxH | F | GCGCAAAGCAAGCTATGTAAA | Amplifies a 172 bp region within *lpxH* |
| lpxH | R | TTTGAACCAGTCGCCAAGTA |  |

**Table S4: Raw Growth, Biofilm and Biofilm:Growth Ratios in *Francisella* Pyrimidine Pathway Mutants in MMHB.**

|  | **Mutant Name** | **Locus Tag** | **Growth (OD_600_)** | | | **Biofilm (OD_590)_** | | | **BF:G Ration (OD_590_:OD_600_)** | | |
| --- | --- | --- | --- | --- | --- | --- | --- | --- | --- | --- | --- |
| WT |  |  | 0.41 | 0.41 | 0.31 | 0.20 | 0.13 | 0.11 | 0.48 | 0.32 | 0.35 |
| *qseC* | tnfn1_pw060418p04q101 | FTN_1617 | 0.40 | 0.40 | 0.33 | 0.05 | 0.04 | 0.04 | 0.13 | 0.09 | 0.12 |
| *carA-1* | tnfn1_pw060328p04q165 | FTN_0021 | 0.38 | 0.36 | 0.29 | 0.23 | 0.16 | 0.16 | 0.60 | 0.43 | 0.56 |
| *carA-2* | tnfn1_pw060510p02q160 | FTN_0021 | 0.12 | 0.10 | 0.10 | 0.04 | 0.09 | 0.10 | 0.36 | 1.00 | 0.96 |
| *carB-1* | tnfn1_pw060323p08q120 | FTN_0020 | 0.38 | 0.34 | 0.23 | 0.12 | 0.11 | 0.10 | 0.31 | 0.32 | 0.44 |
| *carB-2* | tnfn1_pw060328p01q118 | FTN_0020 | 0.12 | 0.12 | 0.08 | 0.05 | 0.12 | 0.13 | 0.44 | 0.97 | 1.67 |
| *pyrB-1* | tnfn1_pw060510p01q109 | FTN_0019 | 0.39 | 0.38 | 0.27 | 0.16 | 0.19 | 0.18 | 0.40 | 0.50 | 0.66 |
| *pyrB-2* | tnfn1_pw060323p03q119 | FTN_0019 | 0.13 | 0.29 | 0.16 | 0.01 | 0.10 | 0.10 | 0.12 | 0.35 | 0.63 |
| *pyrC-1* | tnfn1_pw060328p06q113 | FTN_0024 | 0.37 | 0.35 | 0.19 | 0.25 | 0.14 | 0.13 | 0.66 | 0.41 | 0.75 |
| *pyrC-2* | tnfn1_pw060510p02q166 | FTN_0024 | 0.42 | 0.40 | 0.22 | 0.15 | 0.18 | 0.19 | 0.36 | 0.45 | 0.89 |
| *pyrD-1* | tnfn1_pw060323p03q144 | FTN_0036 | 0.39 | 0.37 | 0.34 | 0.23 | 0.17 | 0.19 | 0.58 | 0.46 | 0.57 |
| *pyrD-2* | tnfn1_pw060510p04q195 | FTN_0036 | 0.40 | 0.33 | 0.33 | 0.18 | 0.11 | 0.15 | 0.45 | 0.34 | 0.47 |
| *pyrE* | tnfn1_pw060510p01q127 | FTN_0529 | 0.06 | 0.11 | 0.09 | 0.55 | 0.27 | 0.37 | 9.15 | 2.40 | 3.94 |
| *pyrF-1* | tnfn1_pw060323p04q176 | FTN_0035 | 0.40 | 0.31 | 0.31 | 0.18 | 0.26 | 0.33 | 0.46 | 0.81 | 1.08 |
| *pyrF-2* | tnfn1_pw060510p01q129 | FTN_0035 | 0.39 | 0.35 | 0.30 | 0.15 | 0.09 | 0.10 | 0.38 | 0.27 | 0.34 |
| *lpxH* | tnfn1_pw060328p04q130 | FTN_0528 | 0.40 | 0.37 | 0.34 | 0.24 | 0.22 | 0.23 | 0.60 | 0.59 | 0.66 |
| *upp-1* | tnfn1_pw060328p08q110 | FTN_0628 | 0.3 | 0.2 | 0.2 | 0.1 | 0.1 | 0.2 | 0.27 | 0.46 | 0.77 |
| *upp-2* | tnfn1_pw060510p04q190 | FTN_0628 | 0.5 | 0.4 | 0.4 | 0.2 | 0.2 | 0.2 | 0.48 | 0.37 | 0.59 |
| *lysR-like-1* | tnfn1_pw060328p07q186 | FTN_0031 | 0.4 | 0.4 | 0.33 | 0.3 | 0.3 | 0.3 | 0.6 | 0.8 | 0.8 |
| *lysR-like-2* | tnfn1_pw060419p03q158 | FTN_0031 | 0.3 | 0.4 | 0.5 | 0.2 | 0.3 | 0.6 | 0.7 | 0.7 | 1.3 |

**Figure S4: Normalized Biofilm:Growth Ratio of *carABpyrBCDEF* Mutants in TSB-C verus MMHB.** All transposon mutants are represented as the biofilm:growth ratio, normalized to the wild type (WT) of their respective baseline growth conditions (i.e. all TSB-C data presented was normalized to the WT grown in TSB-C), where WT is equal to 1.

**Table S5: BPROM promoter analysis of carA 5’UTR.** The *carA* promoter region spanning 425 nucleotides upstream of the *carA* ATG site was analyzed with the BPROM v2.0 software (Softberry Inc.) [42] using default parameters for *Escherichia coli* promoter prediction. (GenBank *Francisella tularensis* subsp. *novicida* U112, CP009633.1:197157-197581). Predictions with scores above 0.8 were considered potential regulatory regions. Putative P1 and P2 −35/−10 elements were identified at position 88 and 385. In additional analysis, ProPr identified two potential σ^54^ binding sites, AACTATAAT and TACAATACT, shown in dark grey with underscore.

| **CP009633.1:197157-197581: *Francisella tularensis subsp. novicida* U112** | | |
| --- | --- | --- |
| Length of sequence- 425 | Threshold for promoters - 0.20 | Number of predicted promoters - 3 |
| Promoter Pos: 88 | LDF- 8.76 |  |
|  | -10 box at pos. 73 TTATAGATT | Score: 38 |
|  | -35 box at pos. 50 TTTAAA | Score: 41 |
| Promoter Pos: 385 | LDF- 1.77 |  |
|  | -10 box at pos. 369 TTTCAAAAT | Score: 53 |
|  | -35 box at pos. 355 ATGACA | Score: 36 |
